# Frugivore richness poorly predicts seed dispersal effectiveness under climate change

**DOI:** 10.1038/s41598-026-43964-0

**Published:** 2026-04-29

**Authors:** Eduardo D. B. Rigacci, Wesley Rodrigues Silva, Michiel P. Boom, W. Daniel Kissling

**Affiliations:** 1 Programa de Pós-Graduação Em Ecologia, Instituto de Biologia, Universidade Estadual de Campinas, Campinas, Brazil; 2 Departamento de Biologia Animal, Instituto de Biologia, Universidade Estadual de Campinas, Campinas, Brazil; 3 Dutch Centre for Field Ornithology, PO Box 6521, 6503 GA Nijmegen, The Netherlands; 4 Institute for Biodiversity and Ecosystem Dynamics (IBED), University of Amsterdam, 1090 GE Amsterdam, The Netherlands

**Keywords:** Ecology, Ecology, Plant sciences

## Abstract

Animal-mediated seed dispersal underpins forest maintenance, yet climate change threatens this mutualism by reshaping plant-frugivore overlap. Most projections assume that frugivore richness and binary interactions adequately capture seed dispersal success, overlooking the unequal quantitative and qualitative contributions of individual species. Here, we combine estimates of seed dispersal effectiveness (SDE) with species distribution models to assess climate change impacts on seed dispersal of two keystone trees—the jussara palm (*Euterpe edulis*) and silver cecropia (*Cecropia hololeuca*)—in the semideciduous Atlantic Forest. We show that frugivore richness is a poor predictor of future dispersal outcomes: across approximately 60% of projected plant ranges, richness either under- or over-estimated seed dispersal and germination potential. This mismatch was species-specific. For the jussara palm, richness explained < 13% of the variance in climate-driven changes in both dispersal quantity and germination success, whereas for silver cecropia, richness aligned only with germination outcomes. Consequently, projected changes in frugivore assemblages and seed dispersal function can diverge under climate change. Our findings demonstrate that richness-based metrics can misrepresent climate risks to plant-frugivore mutualisms and that incorporating functional measures of SDE yields more accurate forecasts of plant recruitment potential and ecosystem-level disruption—essential for predicting ecosystem resilience in a warming world.

**Supplementary Information:**

The online version contains supplementary material available at 10.1038/s41598-026-43964-0.

## Introduction

Animal-mediated seed dispersal underpins tropical forest dynamics, with up to 90% of woody plant species relying on vertebrate frugivores for dispersal^1^. In exchange for nutritional rewards from fruit pulp or arils, frugivores transport seeds to suitable sites, enhancing metapopulation connectivity, reducing intraspecific competition, escaping species-specific seed predators^2–4^, increasing germination success^5^, and facilitating climate-driven niche tracking^6^. Climate change threatens this mutualism by reshaping species distributions, potentially generating novel interactions through the arrival of disturbance-adapted or the persistence of climate-resilient species while simultaneously eroding long-standing plant-frugivore relationships. Such redistribution may decouple plants from their dispersers^7,8^, altering not only whether seed dispersal occurs, but also which species provide this service and how effectively it is delivered. Despite the central role of seed dispersal in tropical ecosystems, the functional consequences of climate-driven changes in species overlap remain poorly understood. This gap arises largely because most forecasts rely on frugivore richness or binary plant-disperser interactions as proxies for dispersal success (e.g., refs^9–12^), overlooking the strongly unequal quantitative and qualitative contributions of individual frugivore species^13^. As a result, our ability to predict how climate change will reshape, and thus the resilience of tropical ecosystems, remains limited.

Focusing on the functional roles of individual species rather than community-level metrics offers a more mechanistic perspective on how climate change may alter seed dispersal processes^13^. Measures of taxonomic diversity such as species richness often obscure functional variation: while some frugivores are functionally redundant, others provide unique and irreplaceable dispersal services^14,15^, such that the loss of a single key species can cause a disproportionate decline in dispersal function^16^. Frugivore richness is therefore expected to track dispersal outcomes when contributions are relatively evenly distributed among species, but to become a poor proxy when dispersal function is strongly skewed toward a few dominant contributors. Seed dispersal effectiveness (SDE) captures this variation by integrating quantitative components (the number of seeds dispersed) with qualitative components (the probability that dispersed seeds successfully establish)^17,18^. Although this framework has been widely applied in empirical, site-based studies, often in local or oceanic systems^19,20^, to quantify species-specific dispersal roles, both components of SDE are shaped by species traits such as body size, gape limitation, and seed-handling behaviour, leading to large interspecific differences in functional contributions^17,21^. Despite growing recognition that incorporating biotic interactions can improve biodiversity forecasts^22,23^, functional dimensions of SDE remain rarely integrated into spatially explicit projections of how climate change will affect animal-mediated plant dispersal.

Incorporating SDE into biodiversity forecasts remains challenging because empirically quantifying both its quantitative and qualitative components across interaction networks is logistically demanding^24^. Such data are scarce, as they require intensive field sampling^25,26^, including frugivory observations, faecal or regurgitation analyses, and germination experiments^17^. The substantial time investment involved often constrains datasets geographically and limits their ability to capture spatial variation in SDE, which can arise from multiple sources such as habitat structure, plant crop size, and local frugivore assemblages. Nevertheless, when interpreted with careful attention to context dependence, SDE provides a powerful mechanistic framework for quantifying species-specific contributions to seed dispersal^17,27^. Integrating field-based estimates of SDE with species distribution models under current and future climates makes it possible to evaluate how plant-frugivore interactions shift across space and time. This approach moves beyond richness-based proxies to reveal the functional consequences of biodiversity change, offering a more predictive understanding of how climate change may alter seed dispersal dynamics, plant recruitment, and ultimately the resilience of tropical ecosystems.

Here, we integrate field-based empirical estimates of SDE—derived from direct frugivory observations, feeding trials, and germination experiments—with species distribution models to test how function-based approaches modify richness-based forecasts of seed dispersal under climate change. We focus on two keystone tree species of Brazil’s Atlantic Forest: the silver cecropia (*Cecropia hololeuca*) and the jussara palm (*Euterpe edulis*), both of which fruit during the winter dry season, when resources are scarce^28,29^, and therefore represent critical food sources for a wide range of vertebrate frugivores. As a result, these species occupy highly central positions in seed dispersal networks across fragmented forests, and substantial empirical evidence indicates that many interacting frugivores act as legitimate seed dispersers (i.e., they do not destroy the seeds)^28,30–32^. The two systems also offer complementary advantages for field-based study: the jussara palm is among the best-documented tropical plant-frugivore systems in terms of species life-history traits and geographic distributions^33,34^, while silver cecropia is readily identifiable in forest fragments by its distinctive silvery foliage^29^. Our study is set within the Atlantic Forest, a global biodiversity hotspot characterized by high endemism and severe habitat loss^35^, where semideciduous forests are particularly vulnerable due to strong seasonal bottlenecks in fruit availability^36,37^.

Focusing on these keystone plant species, we address three main questions: (1) how frugivores differ in their quantitative and qualitative contributions to seed dispersal, (2) how climate change will alter the composition and spatial redistribution of seed dispersers, and (3) to what extent do richness-based forecasts capture climate-driven changes in seed dispersal effectiveness. Based on the pronounced functional heterogeneity among frugivores and their contrasting climatic tolerances, we formulated three expectations. First, we predicted strong interspecific variation in dispersal contributions, with a small subset of species disproportionally contributing to both quantitative and qualitative components of seed dispersal^21,38^. Second, we expected climate change to reduce plant-frugivore co-occurrence overall, while allowing the persistence of some climate-resilient dispersers^9,39^. Third, we anticipated that richness-based forecasts would frequently diverge from SDE-based projections, because the loss or persistence of a few highly effective dispersers can drive disproportionate functional declines or buffering effects^13,15,40,41^.

## Results

### Quantitative and qualitative contributions to seed dispersal

**Fig. 1:**
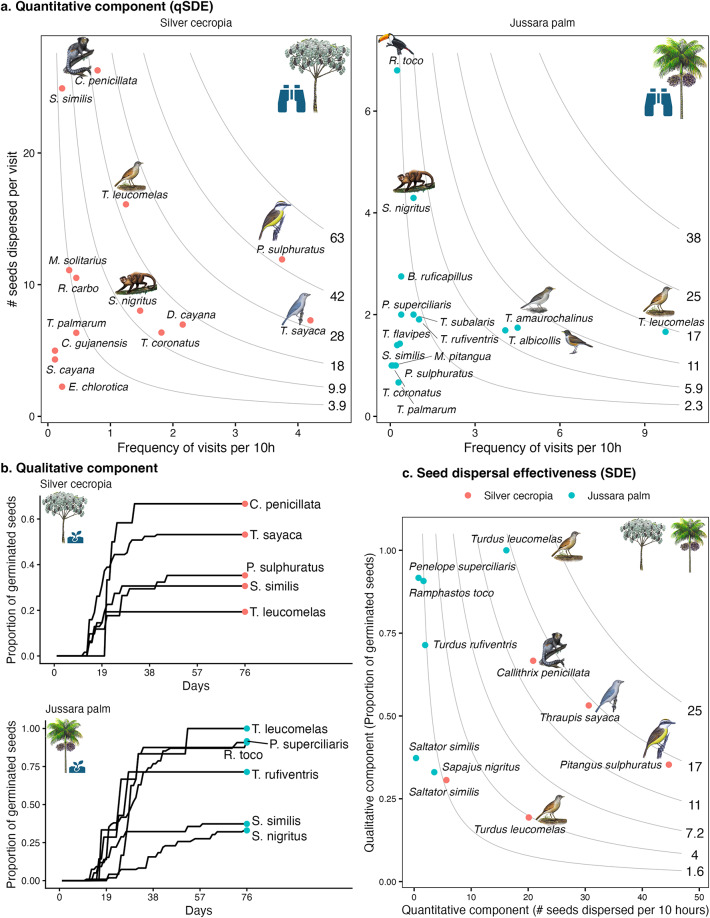
Frugivore contributions to quantitative, qualitative and overall seed dispersal effectiveness (SDE) for two keystone trees in the Atlantic Forest. (**a**) Quantitative component: frequency of visits (x-axis) versus seeds dispersed per visit (y-axis), with isoclines indicating equal dispersal output. (**b**) Qualitative component: cumulative germination proportion (y-axis) of seeds over time (x-axis) after gut passage through different frugivores. (**c**) Seed dispersal effectiveness: product of quantitative (x-axis) and qualitative (y-axis) components, with isoclines showing equal effectiveness. Key dispersers differ strongly between silver cecropia (*Cecropia hololeuca*) and jussara palm (*Euterpe edulis*), highlighting the disproportionate role of a few species such as the black-pencilled marmoset (*Callithrix penicillata*), sayaca tanager (*Thraupis sayaca*), and the pale-breasted thrush (*Turdus leucomelas*). See Supplementary Tables 1 and 2 for supporting data.

Based on 350 h of focal tree observations in semideciduous Atlantic Forest fragments, we documented 23 frugivore species (21 birds, two mammals) dispersing seeds: 14 species (12 birds, two mammals) for silver cecropia and 15 (14 birds, one mammal) for jussara palm. Using focal tree observations, we quantified the quantitative component of seed dispersal effectiveness (qSDE) as the number of seeds dispersed per frugivore species, based on visitation frequency and seeds consumed per visit. Small (< 50 g) and medium-sized (> 50 g) birds dominated visits, while larger frugivores (> 100 g) were rarer but dispersed more seeds per visit. Feeding trials and germination experiments revealed strong interspecific differences in qualitative seed dispersal contributions (Fig. 1b). For silver cecropia, mean germination after gut passage was ~ 40% (vs. 9% for controls), and ~ 70% for jussara palm (vs. 18% for controls), with some dispersers exceeding 90%. Germination data were available for 35% of dispersers of silver cecropia and 40% for jussara palm. Combining quantitative and qualitative components, SDE estimates highlighted a few key species: the black-pencilled marmoset (*Callithrix penicillata*), sayaca tanager (*Thraupis sayaca*), and great kiskadee (*Pitangus sulphuratus*) for silver cecropia, and the pale-breasted thrush (*Turdus leucomelas*) as the dominant disperser of jussara palm (Fig. 1c).

### Climate change effects on species redistributions

Using ensemble forecasts under moderate and business-as-usual climate scenarios, we projected current and future distributions of the two keystone trees and their frugivores in the semideciduous Atlantic Forest. Silver cecropia currently occupies ~ 20% of the region (164,644 km^2^) and jussara palm ~ 54% (512,801 km^2^), with both predicted to undergo marked range contractions. By 2070, silver cecropia is expected to lose 21% and 34% of its range under a moderate and business-as-usual climate scenario, respectively, while the jussara palm is projected to contract its range by 14% and 24% under the same scenarios (Supplementary Table 3). Frugivores are also predicted to face widespread range contractions: current ranges have an average size of 384,637 ± 114,901 km^2^ and are projected to contract by 23 ± 21% under moderate and 37 ± 17% under business-as-usual scenarios (Supplementary Table 3).

### Richness-based forecasts of plant-disperser interactions

Frugivore richness and spatial overlap with both keystone plants are projected to decline under future climates. Within their current ranges, silver cecropia co-occurs with an average of 9 ± 4 frugivores per grid cell and jussara palm with 8 ± 4. Climate change is expected to reduce this by ~ 1–2 species per cell, leaving ~ 7 dispersers on average for each plant (Supplementary Fig. 1). Spatial overlap (i.e., the area simultaneously predicted as suitable for each plant-animal pair) also contracts substantially. At present, frugivores associated with the silver cecropia overlap with 67 ± 16% of its suitable range, and those of the jussara palm with 56 ± 13% of its range (Supplementary Table 4). Although we project some gains in overlap, particularly in the southern portion of the semideciduous forest, overall projections indicate a net reduction of 25 ± 16% (moderate) to 41 ± 13% (business-as-usual) for silver cecropia, and 26 ± 13% to 39 ± 14% for the jussara palm (Supplementary Fig. 1 and Supplementary Table 4).

### Function-based forecasts of plant-disperser interactions

**Fig. 2:**
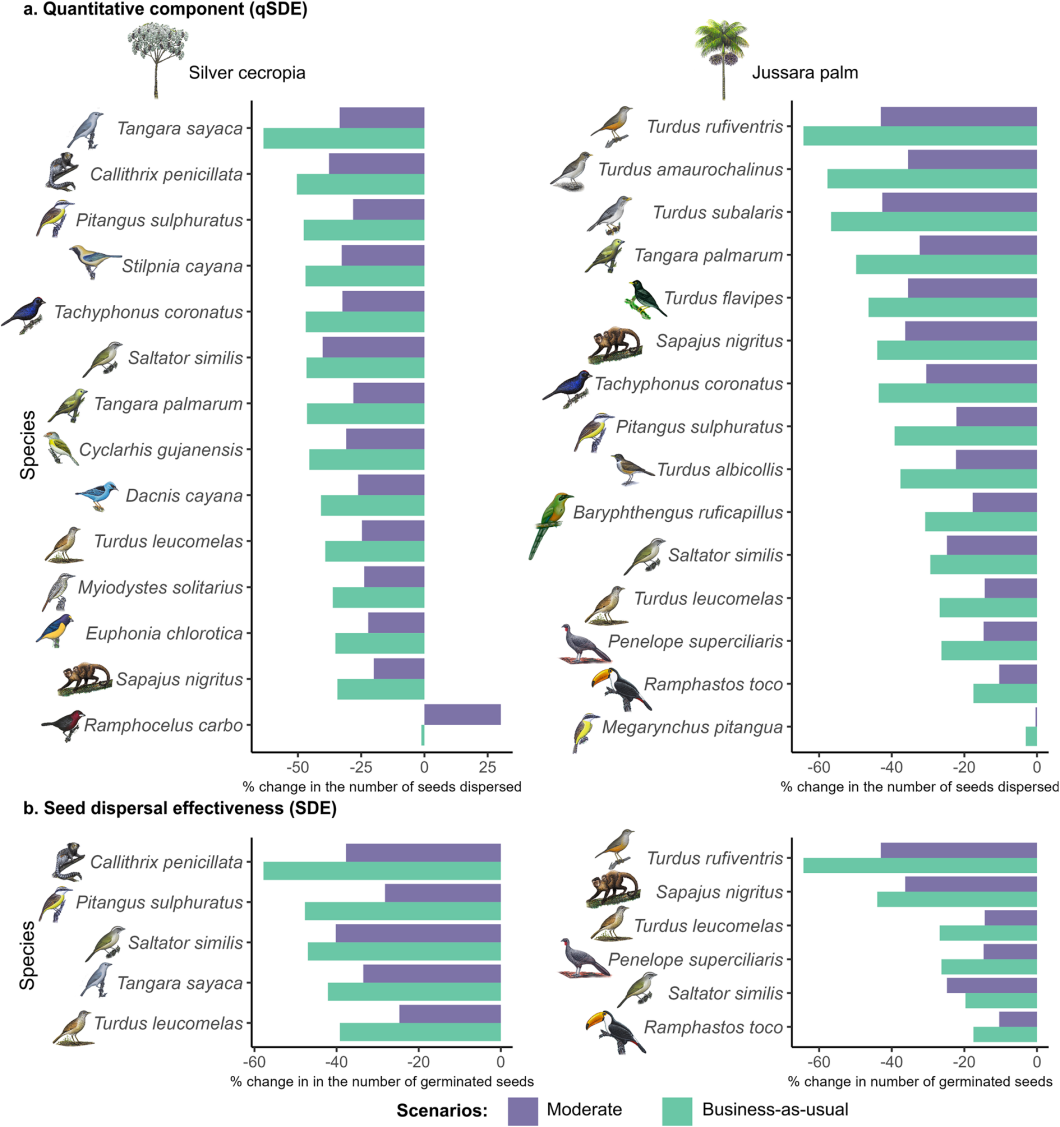
Climate-driven changes in frugivore contributions to seed dispersal of two keystone trees in the semideciduous forest of the Atlantic Forest. (**a**) Percentage change in the quantitative component (qSDE; number of seeds dispersed) across the geographic ranges of silver cecropia (*Cecropia hololeuca*, left) and jussara palm (*Euterpe edulis*, right) for each frugivore relative to current levels. (**b**) Percentage change in overall seed dispersal effectiveness (SDE; number of seeds germinating after gut passage) for the subset of frugivores with available data. Results are shown for both moderate and business-as-usual climate scenarios, highlighting widespread reductions in dispersal function and the disproportionate vulnerability of key frugivores.

By combining empirical qSDE and SDE scores with projected frugivore distributions, we estimated climate impacts on species- and community-level contributions of frugivores to seed dispersal. Nearly all frugivores are projected to reduce their quantitative and qualitative roles under future climates. The only exception is the silver-beaked tanager (*Ramphocelus carbo*), which under a moderate climate change scenario is forecasted to increase its quantitative component by nearly 30% (Fig. 2; Supplementary Tables 5–6). Across their predicted ranges, based on ensemble-mean projections, silver cecropia is expected to experience a ~ 37% decline in dispersed seeds relative to current conditions (− 28% under moderate and − 45% under business-as-usual scenarios), while the jussara palm faces a ~ 30% reduction (− 23% and − 37%, respectively) (Supplementary Fig. 2 and Supplementary Tables 5–6). Germination outcomes follow a similar trend, with declines of ~ 37% for silver cecropia (− 32% to − 41%) and ~ 22% for jussara palm (− 16% to − 28%) (Supplementary Figs. 3–4 and Supplementary Tables 5–6). Importantly, the magnitude of projected losses differs between the quantitative and qualitative components, indicating that species-level contributions decline unevenly once germination success is incorporated. For example, some dispersers experience strong reductions in the number of seeds dispersed, but their overall functional contribution declines even more sharply when seed germination is considered (e.g., the black-pencilled marmoset on silver cecropia).

### Decoupling frugivore richness and seed dispersal function

**Fig. 3:**
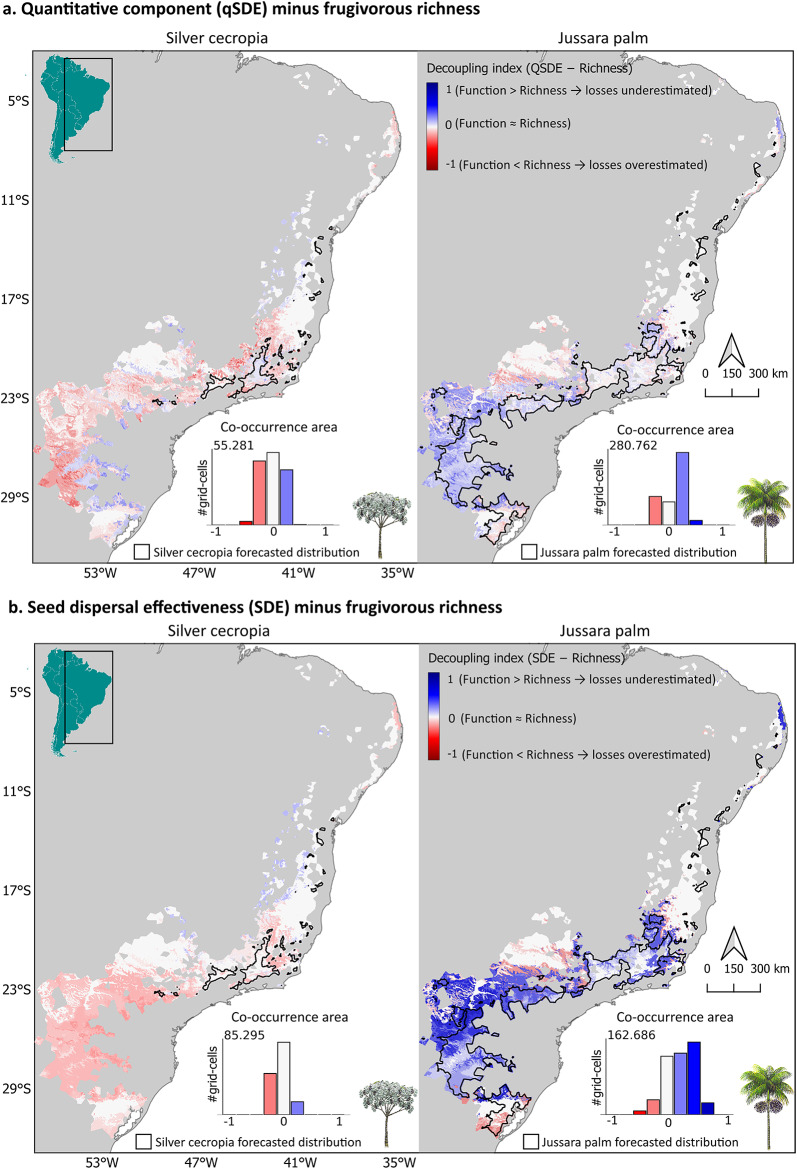
Decoupling of frugivore richness and functional seed dispersal under climate change. Maps show the spatial mismatches between interaction richness and two functional metrics of seed dispersal under a business-as-usual scenario: (**a**) quantitative seed dispersal (qSDE) and (**b**) seed dispersal effectiveness (SDE). Left panels show silver cecropia (*Cecropia hololeuca*); right panels show jussara palm (*Euterpe edulis*). The decoupling index (function minus frugivore richness) ranges from -1 to + 1, where negative values (red) indicate that richness overestimates function, zero (grey) indicates agreement, and positive values (blue) indicate that richness underestimates function. Black outlines denote projected future plant distributions. Insets show frequency distributions of decoupling index values within each plant’s forecasted area of co-occurrence with frugivores (in km^2^).

Richness-based forecasts failed to capture spatial patterns of seed dispersal function—both qSDE (seeds dispersed) and SDE (seeds germinated)—for silver cecropia and jussara palm, which showed contrasting decoupling patterns (Fig. 3 and Supplementary Table 7). Under the business-as-usual scenario, mismatches occurred across most of each species’ range: 63% (qSDE) and 43% (SDE) for silver cecropia, and 60% (qSDE) and 65% (SDE) of jussara palm range. For silver cecropia, mismatches were bidirectional. Richness overestimated qSDE in ~ 31–34% of the range, underestimated it in ~ 28–32%, and matched function in ~ 36–37%. For SDE, richness aligned more often (57–63%) but still showed substantial overestimation (20%–32%) or underestimation (10–17%). By contrast, jussara palm showed consistent unidirectional decoupling, with function exceeding richness-based predictions across most of its range (qSDE: ~ 59–60%, SDE: ~ 65%).

Linear models revealed contrasting strengths of the decoupling between richness and function (Supplementary Table 8). For silver cecropia, richness strongly predicted SDE under both scenarios (moderate: R^2^ = 0.95–0.96; slopes ≈ 0.9) and explained qSDE under business-as-usual conditions (R^2^ = 0.62). However, its predictive power collapsed for qSDE under the moderate scenario (R^2^ = 0.06). By contrast, richness consistently failed to predict either qSDE or SDE for jussara palm (R^2^ ≤ 0.13 across scenarios). Thus, differences in the richness–function relationship reflect how evenly seed dispersal contributions are distributed among frugivores, with stronger functional asymmetries leading to weaker correspondence between richness and seed dispersal effectiveness.

## Discussion

Our study shows that climate change is likely to disrupt vertebrate-mediated seed dispersal in the semideciduous Atlantic Forest in ways that species richness alone fails to capture. By combining empirical measures of seed dispersal function (qSDE and SDE) with species distribution forecasts, we show that richness consistently misrepresented functional outcomes—both the number of seeds dispersed and those successfully germinating—across large portions of the projected future ranges of two keystone tree species. The magnitude and direction of this mismatch varied across tree species and scenarios, revealing strong context dependence in richness-function relationships. Together, these results indicate that functional contributions are unevenly distributed among frugivores and that richness-based metrics can obscure critical changes in mutualistic performance, emphasizing the need for functionally explicit approaches when anticipating climate-driven impacts on plant-frugivore interactions.

Our results highlight the disproportionate role of small-bodied generalists in contemporary seed dispersal, reflecting pervasive defaunation in semideciduous Atlantic Forest fragments^42^, where larger frugivores are locally or functionally extinct^43^. This compositional shift elevates the importance of thrushes and tanagers^44^ and may even have driven evolutionary responses in plants, such as reduced seed size in the jussara palm^28^. Across frugivore species, we detected marked differences in both seed removal and germination effects, with contrasting functional patterns between the two focal plants. For silver cecropia, its small seeds (~ 2 mm) allowed high numbers of seeds numbers to be dispersed per visit (high qSDE), while germination success varied little among species, rendering overall SDE largely quantity-driven. This pattern is consistent with systems dominated by small-seeded plants interacting with generalized frugivore assemblages^21,38,45^. In contrast, the larger fruits of the jussara palm impose constraints on both disperser identity and performance: gape-size limitations restrict which species can act as dispersers, longer handling times reduce the number of seeds dispersed per visit^46^, and satiation or seed ballast effects can further depress visitation rates^46,47^. As a result, jussara seed dispersal exhibited pronounced interspecific variation in both quantitative and qualitative components. Toucans and guans visited less frequently and dispersed relatively fewer seeds but achieved high germination success, black capuchins dispersed many seeds with low germination success, and thrushes combined both high seed removal with high germination. These contrasts generate a steep functional hierarchy in which a small subset of frugivores disproportionately determines SDE through either quantitative effects, qualitative effects, or both^48^. Moreover, because our SDE estimates do not incorporate seed deposition patterns, the functional importance of large frugivores like toucans and guans is likely underestimated, particularly given their capacity to mediate long-distance seed dispersal and connect forest types across their extensive movement ranges^49^.

By 2070, the semideciduous Atlantic Forest is projected to experience warming of up to 3.5 °C and increased aridity in the central and northern regions^50,51^, with potentially severe consequences for keystone plants and their associated frugivore assemblages. Our forecasts indicate pronounced range contractions for both silver cecropia and the jussara palm, coupled with substantial losses of canopy-dependent frugivores such as primates, which are unlikely to track shifting climates across a highly fragmented and low-permeability landscape^52,53^. Beyond the direct effects of range loss, which alone poses a major conservation challenge^54^, climate change is expected to destabilize plant-frugivore mutualisms^9,39^, as evidenced here by sharp declines in spatial co-occurrence and local disperser richness. Crucially, when seed dispersal effectiveness was incorporated into species distribution projections, these emerging spatial mismatches translated into large reductions in both seed dispersal quantity and successful germination for both plant species. Over medium to long time scales, such declines in dispersal function are likely to compromise recruitment, gene flow, and ultimately the persistence of these keystone plants, further exacerbating the vulnerability of the already imperilled semideciduous Atlantic Forest^55^.

We found that frugivore richness alone misrepresented future seed dispersal and germination potential across approximately 60% of the projected ranges of both plant species. This decoupling likely arises from the interaction between climate-driven shifts in plant-frugivore co-occurrence—determined by species-specific climate sensitivities and resulting spatial overlap—and the highly uneven distribution of functional contributions among frugivores, as captured by applying species-specific functional weights applied to binary presence maps. These patterns reinforce the need for trait- and function-based frameworks to more accurately anticipate ecosystem responses to climate change^8,13,56^. In regions where dispersal function exceeded expectations based on richness, a small number of climate-resilient species contributed disproportionately to dispersal and germination, buffering functional losses despite declining richness. In contrast, where richness overestimated function, the species most sensitive to climate change were often those with the greatest dispersal effectiveness, and their loss left functionally weaker dispersers unable to compensate. Consequently, richness-based metrics can obscure profound shifts in functional dynamics, a pattern also reported for long-distance seed dispersal processes^6,16,41^. Together, these results highlight a critical conservation implication: taxonomic presence alone is an unreliable proxy for ecosystem functioning under climate change.

The strength of the richness-function decoupling differed markedly between the two plant species. For silver cecropia, decoupling was weak: frugivore richness reliably predicted qSDE under the business-as-usual scenario and SDE under both climate scenarios. This reflects relatively even functional contributions across dispersers that are largely independent of species-specific climatic vulnerability^13,57^. Although per-visit seed dispersal scales with beak size in birds and jaw size in primates, seed dispersal is dominated by small birds, while primates interact infrequently, resulting in broadly additive and weakly hierarchical contributions. Importantly, the most effective dispersers were not disproportionately climate-sensitive, and under moderate climate change, species turnover was concentrated among low-contribution frugivores, buffering overall function losses. In contrast, for the jussara palm, richness was a poor predictor of both qSDE and SDE. Here, seed dispersal function was concentrated in a small subset of species, particularly climate-resilient thrushes. Notably, the Pale-breasted Thrush combined high seed dispersal with high germination success while experiencing only modest projected range loss. Larger dispersers such as toucans and guans, also projected to retain substantial spatial overlap with the palm, may further reinforce this pattern. Consequently, whereas cecropia dispersal appears to be buffered by diffuse contributions across a broad frugivore assemblage, jussara palm dispersal depends disproportionately on a few climate-resilient species, amplifying richness–function decoupling under climate change.

Our projections are necessarily shaped by the scope of the empirical data, the ecological information available for each species, and the modelling assumptions adopted. We acknowledge that our field-based observations may not capture the full assemblage of potential dispersers for the two focal plants, particularly nocturnal and ground-dwelling species. Moreover, although seed dispersal effectiveness was quantified over two years and multiple sites to incorporate spatial and temporal variation, additional sources of variability, such as interannual fluctuations in fruit production and changes in frugivore abundance, may further influence visitation rates and germination outcomes. Accordingly, the spatially explicit seed dispersal metrics presented here should be interpreted as relative indices of potential dispersal and germination capacity rather than estimates of realized seed flux.

Functional losses may be partly buffered by dispersers not included in our analyses, especially where such species perform similar functional roles and are less vulnerable to climate change^56^. This form of compensation is more plausible for silver cecropia, whose small seeds attract a broad range of generalist dispersers^29^, including bats and additional bird species^58^, than for the jussara palm, whose larger seeds strongly constrain disperser identity^28^ and exclude many species already functionally extinct in fragmented Atlantic Forests (e.g., tapirs)^28,43^. Indeed, bat-mediated dispersal has been shown to enhance germination probabilities in some *Cecropia* species^59^. While trophic rewilding could, in principle, restore lost dispersal functions^43,60,61^, the extent to which ecosystem functioning can be recovered—whether via overlooked, novel, or reintroduced dispersers—ultimately depends on the functional roles these species perform rather than their taxonomic presence alone. A remaining and more general limitation is the scarcity of empirical seed dispersal effectiveness data^20,25,26^, which continues to constrain robust functional projections across species and ecosystems. Expanding such measurements is therefore critical for improving forecasts of how biodiversity loss and climate change will reshape ecosystem functioning and the services it underpins.

By integrating empirical measures of seed dispersal effectiveness with species distribution models, we show that climate-driven changes in frugivore richness can diverge sharply from functional outcomes. Critically, both the magnitude and direction of this divergence depend on how unevenly dispersal function is distributed among frugivores: when contributions are relatively homogeneous, richness and function tend to covary spatially, whereas strong functional asymmetries cause richness to misrepresent functional vulnerability. These results underscore that improving biodiversity forecasts under climate change requires moving beyond taxonomic metrics to explicitly incorporate interaction processes—such as visitation rates, fruit removal, and germination success—that directly determine ecosystem functioning. Although such data remain limited for most plant-animal interactions, embedding functional information into predictive frameworks offers a tractable path to anticipating interaction loss and prioritizing monitoring and conservation actions an increasingly dynamic and uncertain world.

## Methods

Our workflow (Supplementary Fig. 5) combined empirical and secondary data—including frugivore observations, occurrence records, environmental variables, feeding trials, and germination experiments—to model species distributions and quantify seed dispersal (qSDE and SDE). Richness-based forecasts were derived from plant-frugivore co-occurrence, while function-based forecasts integrated qSDE and SDE with species distributions to estimate the potential number of seeds dispersed and germinated under current and future scenarios. Two keystone tree species of Brazil’s Atlantic Forest and their frugivore assemblages were included.

### Keystone tree species

We focused on two keystone tree species (Supplementary Fig. 6). The silver cecropia (*Cecropia hololeuca*), a 6–12 m tall tree of primary and secondary forests, produces infructescences consisting of three catkins bearing numerous small seeds^29^, ripening from June to November^62^. The jussara palm (*Euterpe edulis*), a 8–12 m tall single-stemmed palm of old-growth forest, bears single-seeded, round fleshy drupes (13.5 ± 1.3 mm diameter)^28^ that ripen from April to August^62^.

### Study area

The semideciduous forest, a formation within the Atlantic Forest of South America, is characterized by strong seasonality with a rainy period (October–March) and a dry period (April–September)^63^. It extends from Brazil to Paraguay and Argentina (8°–30°S, 35°–57°W), spanning habitats from alluvial and (sub)tropical to montane and submontane, along an elevational gradient up to 1200 m^64^. As part of the Atlantic Forest biodiversity hotspot, the semideciduous forests face intense anthropogenic pressures, including severe deforestation and habitat fragmentation^35^. Land-use change has converted large tracts into cropland, pastures, urban areas, and roads^43,52^, and protection remains scarce, with only ~ 7% under reserves^65^. We selected three field sites in São Paulo, Brazil, that are representative of the current ecological status of semideciduous forest remnants: small in size (< 2000 ha in 60% of the fragments), embedded in human-dominated matrices^52^, and subject to intense defaunation, with larger, forest-specialist frugivores largely being absent or functionally extinct^42,43^. The three field sites were: 1) Estação Ecológica dos Caetetus (22°24′10″S 49°42′04″W; 2178 ha), with a rural matrix where only jussara palm occurs; 2) ARIE Mata Santa Genebra (22°49′18″S 47°06′38″W; 252 ha), a peri-urban fragment where both jussara palm and silver cecropia occur; and 3) Parque Estadual da ARA in Valinhos (23°00′37″S 47°04′06″W; 64 ha), also peri-urban, where only silver cecropia occurs^66^. The two peri-urban sites are ~ 20 km apart and ~ 275 km east of Estação Ecológica dos Caetetus.

### Seed dispersal effectiveness of frugivores

#### Quantitative component

At each site, focal tree observations were used to characterize frugivore-plant interaction networks and to derive the quantitative component of seed dispersal effectiveness (qSDE; Supplementary Fig. 5). Observations were conducted during fruiting seasons across two years: April-August 2021/2022 for jussara palm and July-November 2021/2022 for silver cecropia. We selected trees with unobstructed crowns and ripe fruits (dark purplish drupes for jussara palm, dark brown catkins with peck marks for silver cecropia^67^).

Frugivore visits were recorded during morning (05:00–10:00) and afternoon (16:00–18:00) sessions in 30-min blocks with 5-min breaks. We documented species identity and assessed number of seeds dispersed per visit, defining dispersal events as either (a) seed swallowing or (b) seed carrying away from the parent tree^1^. The frugivore assemblages included in this study were primarily treated as legitimate seed dispersers, that is, species whose main foraging behaviour does not involve systematic seed destruction, at least for the two focal plants, based on our field observations and the available literature (e.g., refs^28,68,69^). We did not record visits by ground-dwelling frugivores due to logistical constraints during field observations. Moreover, in the highly defaunated semideciduous forest context considered here^70^, most ground-dwelling frugivores interacting, with jussara palm fruits are predominantly reported as seed predators rather than legitimate seed dispersers (e.g., rodents)^28^. For this reason, excluding ground frugivores is unlikely to bias our estimates of seed dispersal effectiveness in this system. In total, 48 jussara palm individuals (284 ± 129 min per tree) and 21 silver cecropia individuals (350 ± 136 min per tree) were observed.

Because jussara palm fruits are single-seeded, each fruit dispersed corresponds to one seed. In silver cecropia, however, frugivores consume only portions of catkins containing many small seeds, requiring species-specific estimates of seed intake. For birds, we estimated seeds per peck by calculating beak volume (*BV*) using the cone formula adapted from ref^71^.

1 $$BV = {{1} \mathord{\left/ {\vphantom {{1} 3}} \right. \kern-0pt} 3}\pi r^{{2}} h$$

where *r* = ½ beak width (measured between commissures^72^) and *h* = cone height (culmen length measured from the beak tip to skull base^73^). Measurements were taken on ≥ 10 museum specimens per species at the Museu de Zoologia da Universidade Estadual de Campinas (ZUEC). Seed density was determined from fresh catkins (*n* = 5 trees × 5 catkins), yielding ~ 1 viable seed per 150 mm^3^ of fresh pulp. Dividing *BV* by this value provided species-specific estimates of seeds per peck^31,74^.

For the two primates (Black-horned Capuchin, Black-pencilled Marmoset), which handle and bite catkins^75^, seed intake was estimated using mandible length (*L*), measured in mm from the last molar insertion point to the central incisor (*n* = 5 skull specimens per species; at ZUEC). Catkin dissections (*n* = 5 trees × 5 catkins) indicated ~ 1 seed per 0.9 mm of fruit length. Thus, dividing *L* by 0.9 mm yielded seeds per bite.

Finally, the quantitative component of SDE for both plants was calculated as the product of frugivore visitation rate (number of visits per 10 h) and mean number of seeds dispersed per visit^17,18^.

We quantified the sampling completeness of our focal observations using the iNEXT function from the “iNEXT” R package^76^. Each unique plant–frugivore interaction was treated as a “species”, and the number of recorded interaction events (i.e., visits resulting in seed dispersal events) was treated as “abundance”. Sampling completeness (SC) was estimated as the ratio between the observed richness of interactions and the asymptotic interaction richness estimated using a Chao-type estimator (q = 0)^77,78^. We further estimated sample coverage and associated 95% confidence intervals, which quantify the proportion of total interactions captured by the observed data^79^. Because our goal was not to compare sites but to obtain a representative seed dispersal network for the two focal plant species across the semideciduous Atlantic Forest, interaction data were pooled by plant species. However, because sampling effort differed among study areas, we first evaluated sampling completeness separately for each plant-area network to ensure that local interaction networks were adequately sampled. Sampling completeness was consistently high across areas (SC = 0.97 ± 0.03; Supplementary Fig. 7), justifying the pooling of interactions for each focal plant. The pooled networks also showed high completeness (silver cecropia: SC = 0.98; jussara palm: SC = 0.99), indicating that the interactions were well sampled (Supplementary Fig. 7).

In addition, to assess whether the observed frugivore assemblages for each plant are representative of published interaction records in the semideciduous Atlantic Forest, we cross-checked our data against the Atlantic-frugivore database^58^, the most comprehensive seed dispersal interaction dataset currently available for the Atlantic Forest. Because our analyses focus specifically on semideciduous forests, we retained only interaction records involving the two focal plants with complete geographic coordinates (representing 36% of available records for silver cecropia and 70% for jussara palm) and further filtered records to those falling within the semideciduous forest polygon. Within this forest type, only two unique interactions were previously reported for silver cecropia. In contrast, our focal observations overlapped with 4 out of 11 previously recorded disperser species for jussara palm (36%), while documenting 11 additional dispersers not explicitly reported for this forest type (Supplementary Fig. 7).

#### Qualitative component

We estimated the qualitative component of SDE from feeding trials and germination experiments (Supplementary Fig. 5), quantifying the effect of frugivore gut passage on seed germination. For each frugivore, qualitative SDE was calculated as the proportion of germinated seeds relative to the total number of seeds tested per treatment^17,18^.

Feeding trials were conducted in captivity at C*entro de Manejo e Reabilitação de Animais Silvestres* (CEMACAS), a wildlife rehabilitation centre in São Paulo, Brazil, using nine frugivore species (seven birds and two primate species), totalling 55 individuals. For the jussara palm, this included the pale-breasted thrush (*Turdus leucomelas*) (*n* = 4), the rufous-bellied thrush (*Turdus rufiventris*) (*n* = 4), the rusty-margined guan (*Penelope superciliaris*) (*n* = 4), the toco toucan (*Ramphastos toco*) (*n* = 8), the green-winged saltator (*Saltator similis*) (*n* = 4), and the black-horned capuchin (*Sapajus nigritus*) (*n* = 8). Ripe infructescences of silver cecropia were offered to five bird species and one primate species, including the sayaca tanager (*Thraupis sayaca*) (*n* = 3), the great kiskadee (*Pitangus sulphuratus*) (*n* = 3), the green-winged saltator (*Saltator similis*) (*n* = 4), the pale-breasted thrush (*Turdus leucomelas*) (*n* = 3), and the black-pencilled marmoset (*Callithrix penicillata*) (*n* = 10). Despite the constraints of working with captive animals, these trials allowed us to obtain data for approximately 35% of the known disperser assemblage of silver cecropia and approximately 40% of the dispersers of jussara palm. Fruits and infructescences were collected from the field sampling sites during the fruiting season, stored under refrigeration, and used within five days of collection.

At CEMACAS, each frugivore species was housed separately in aviaries, and food was withheld for eight hours prior to the experiments to increase the likelihood of fruit consumption. On experimental days, individuals were exclusively provided with either jussara palm fruits or silver cecropia infructescences, and the two plant species were never offered simultaneously. Water was provided ad libitum throughout the trials. Defecated and regurgitated seeds were collected at the end of each experimental day and immersed in water for 12 h to facilitate separation of seeds from faeces and other biological material. In total, we obtained 609 seeds from gut-passage experiments, comprising 313 jussara palm seeds and 296 silver cecropia seeds. These seeds were subsequently compared with control seeds obtained from intact fruits or infructescences in germination experiments. All seeds and fruits were surface sterilized in a 2.5% sodium hypochlorite solution for 5 min and rinsed thoroughly in distilled water^80^, and sown in sterilized Petri dishes on filter paper. Germination trials were maintained in chambers with alternating 28 °C day/20 °C night temperatures and a 12-h photoperiod, conditions mimicking the field. All Petri dishes were regularly watered. We checked for germination every two days. Germination was defined as the emergence of the germinative bottom in jussara palm^81^ and ≥ 1 mm radicle growth or geotropic movement for silver cecropia^82^.

#### Seed dispersal effectiveness

For frugivore species with data on both quantitative (seeds dispersed per 10 h) and qualitative components (germination proportion), we calculated seed dispersal effectiveness (SDE) as their product (Supplementary Fig. 5)^17,18^. This approach integrates both the number of seeds dispersed and their likelihood of germinating after gut passage, providing a standardized measure of each species’ contribution to plant recruitment. We plotted these values in a seed dispersal effectiveness landscape, a two-dimensional representation in which the quantitative and qualitative components define the axes, and isoclines connect all combinations of these components that yield identical effectiveness values^18,83^.

### Species distribution forecasts

We modelled current and future distributions for all frugivore and plant species to assess potential changes in seed dispersal function.

#### Occurrence records

Frugivore occurrences were obtained from Atlantic birds^84^ and Atlantic primates^85^ datasets, the most comprehensive ones currently available for the Atlantic Forest. Plant occurrences were obtained from the Atlantic frugivore dataset^58^ and the Red List of Threatened Species from the National Center for Flora Conservation (CNCFlora; cncflora.jbrj.gov.br). For frugivores and plants we complemented with records from the Global Biodiversity Information Facility (GBIF, www.gbif.org), iNaturalist (www.inaturalist.org), and the Berkeley Ecoinformatics Engine (Ecoengine, www.ecoengine.berkeley.edu). Taxonomy for birds and primates followed the IUCN Red List (April 2023). We obtained 4,016,636 records, with an average of 160,655 ± 277,945 records per species (range: 361–1,40,4619) (Supplementary Table 9).

#### Data cleaning

Species occurrence records were cleaned using the R package “CoordinateCleaner”^86^ by removing duplicates, incomplete coordinates, records in oceans, museums, herbaria, or centroids of municipal and political polygons, and those > 200 km outside IUCN range boundaries. Records with georeferencing uncertainty ≥ 1 km or collected before 1979 were also excluded to match the spatiotemporal resolution of environmental data. This yielded 662,207 records (mean 26,488 ± 36,017 per species). To reduce spatial autocorrelation, occurrences < 1 km apart were thinned with the R package “spThin”^87^, resulting in 250,652 records (mean 10,026 ± 8630; range: 109–32,914). Overall, 86 ± 8% of initial records were removed (Supplementary Table 9).

#### Predictor variables

Environmental predictors included both climatic and, for plants, soil variables (Supplementary Fig. 5; Supplementary Table 10). Because our models targeted species distributions at biogeographical scales and were projected under future climate scenarios, we did not include fine‐scale vegetation‐structure variables (e.g., canopy cover or NDVI), which are not consistently available as robust future projections and may reduce model transferability at broad spatial extents^88–90^. Present-day climate data were obtained from CHELSA v2.1 (www.chelsa-climate.org; 30 arc sec, ~ 1 km^2^; 1979–2013)^91^, while soil variables (0–30 cm depth) were extracted from SoilGrids v2.0 (https://soilgrids.org/; 30 arcsec, ~ 1 km^2^)^92^. To reduce multicollinearity, we applied the ‘vifcor’ function in the R package ‘usdm’^93^, excluding variables with higher variance inflation factor (VIF) values in variable pairs correlated above *r* = 0.6. This procedure yielded 6–8 predictor variables per species (Supplementary Table 11).

#### Species distribution models

We modelled species ranges using species distribution models (SDMs) with species occurrences and environmental predictors (climate and, for plants, soil variables) in the ‘sdm’ R package^93^. Three machine-learning algorithms were applied: boosted regression trees (BRT)^94^, MaxLike^95^ and random forests (RF)^96^—with 75% of records used for calibration and 25% for evaluation. Each algorithm was run 15 times per species, yielding 45 models. Model accuracy was assessed with the true skill statistic (TSS) and area under the curve (AUC) of the receiver operating characteristic (ROC). Ensemble predictions were generated in the ‘sdm’ R package^93^ using a weighted method approach that gave higher weight to models with greater TSS^97^. Background areas were defined by species-specific bounding boxes based on occurrence extremes, extended by an additional 10° in all directions to include potentially accessible areas for each species^98,99^. Environmental layers were cropped to these backgrounds, and pseudo-absences were sampled in equal number to presences.

#### Projected future species distributions

We forecasted future distributions of plants and frugivores by projecting ensemble SDMs under climate change scenarios from the sixth assessment of the International Panel on Climate Change (IPCC-6, www.ipcc-data.org) using CHELSA v2.1 climate data^91^. We selected the MPI-ESM1-2-HR model^100^, which performs well in the Atlantic Forest^101^, and applied two Shared Socioeconomic Pathways (SSP370 and SSP585) 2041–2070, representing moderate (~ 2.1 °C warming by 2070) and business-as-usual (~ 2.4 °C warming by 2070) trajectories. Soil variables for plants were held constant due to the absence of future edaphic projections. Continuous habitat suitability outputs were converted into binary maps using TSS-maximizing thresholds (Supplementary Table 11), ensuring balanced sensitivity and specificity^102^. Final outcomes were species-level maps of current and future potential distributions under both climate scenarios (Supplementary Fig. 5).

### Forecasting animal-plant interactions under climate change

#### Richness-based forecasts

We used binary SDM outputs for the silver cecropia and jussara palm to evaluate shifts in animal-plant interactions across the semideciduous Atlantic Forest (Supplementary Fig. 5). By overlaying the distributions of frugivores with those of their key food plants, we quantified present and future co-occurrence patterns. From these overlaps, we derived maps of frugivore richness per grid cell (i.e. the number of disperser species potentially interacting with each plant) and calculated the proportion of each plant’s range occupied by each frugivore species, thereby mapping the spatial distribution of potential interactions^103^.

#### Function-based forecasts

To assess functional consequences, we projected changes in the quantitative component of seed dispersal (number of seeds dispersed per grid cell) and, for a subset of frugivores with experimental data, in SDE (number of germinated seeds per grid cell). For each grid cell and frugivore–plant pair, we calculated the quantitative contribution or SDE as

2 $${\mathrm{SD}}_{{\mathrm{P,f}}} = Hs_{{\mathrm{f,C}}} \times {\mathrm{qSDE}}_{{\mathrm{f}}}$$

where *SD*_*P,f*_ is the contribution of frugivore *f* to plant *P* in grid cell *C*, *Hs*_*f,c*_ indicates frugivore presence (0/1), and *qSDE*_*f*_ is the species-specific dispersal quantity or SDE score. The cumulative contribution of the frugivore assemblage in each grid cell was then computed as

3 $$SD_{P,C} = \mathop \sum \limits_{f = 1}^{n} {\mathrm{SD}}_{{\mathrm{P,f}}} { }$$

where *SD*_*P,C*_ is the sum of the quantitative component or SDE of all frugivores *n* for plant *P* in grid cell *C*. By comparing current and future assemblage-level contributions within and beyond each plant’s projected range in the semideciduous part of the Atlantic Forest, we quantified potential climate-driven changes in both dispersal quantity and SDE for these two keystone species (Supplementary Fig. 5).

### Decoupling frugivore richness and seed dispersal function

We tested whether frugivore richness predicts seed dispersal function by comparing standardized maps of frugivore richness, the quantitative component of SDE (qSDE), and seed dispersal effectiveness (SDE). All rasters were rescaled to a 0–1 range using min–max normalization,

4 $$x\prime = \frac{x - \min \left( x \right)}{{\max \left( x \right) - \min \left( x \right)}}$$

to allow comparability among metrics. We then calculated a decoupling index as the difference between function (qSDE or SDE) and richness per grid cell and scenario. Index values range from -1 (richness overestimates function) to + 1 (richness underestimates function), with 0 indicating perfect agreement. For the SDE-based index, richness maps only included frugivores for which quantitative and qualitative data were available from feeding trials and germination experiments.

To quantify the strength of decoupling, we calculated percentage changes in richness, qSDE, and SDE between current and future scenarios for each grid cell. We then fitted linear models with changes in qSDE or SDE as response variables and richness change as the predictors. Analyses were conducted separately for each plant species and climate scenario, restricted to stable areas of plant occurrence (i.e. grid cells suitable under both current and future conditions). Models were fitted using ordinary least squares with robust, heteroskedasticity-consistent (HC3 corrected) standard errors, implemented in the R packages ‘lmtest’^104^ and ‘sandwich’^105^.

## Supplementary Information

Below is the link to the electronic supplementary material.

Supplementary Material 1

## Data Availability

The climatic and soil predictor rasters, as well as the curated species occurrence records used for the analyses, are available via Zenodo at 10.5281/zenodo.17054306 (ref.^83^). Species interaction data underpinning the richness- and function-based maps are provided in Supplementary Tables S1 and S2. The R code used for species occurrence acquisition, cleaning, and spatial thinning, as well as code for species distribution modelling, richness- and function-based predictions, and regression analyses, is available via Zenodo at 10.5281/zenodo.17054306 (ref.^83^).
