# Supplementary material for: Frugivore richness poorly predicts seed dispersal effectiveness under climate change
Source: Sci Rep. 2026 Apr 29;16:13775. doi: 10.1038/s41598-026-43964-0 (PMC13129050; doi:10.1038/s41598-026-43964-0)
Supplement: Supplementary file 1 — Supplementary Material 1 [file 41598_2026_43964_MOESM1_ESM.docx]

**Supplementary information**

**Supplementary figures**


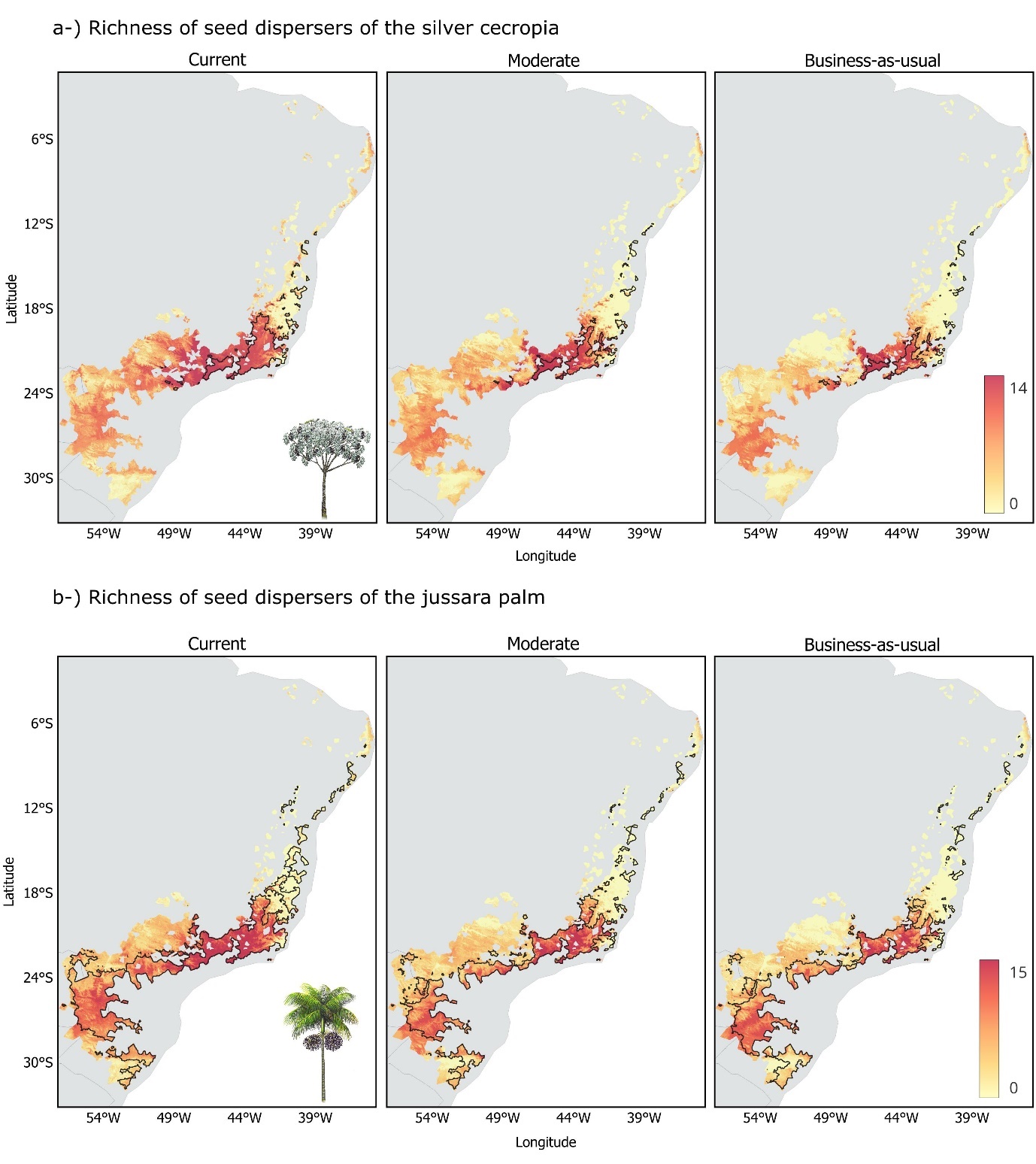


**Supplementary Fig. 1** **| Species richness of frugivores dispersing two keystone plants under current and future climates.** (**a**) Seed dispersers of silver cecropia (Cecropia hololeuca). (**b**) Seed dispersers of jussara palm (Euterpe edulis). Maps depict current richness (left) and projected richness under moderate (middle) and business-as-usual (right) climate conditions. Black contour lines delineate current and forecasted geographic ranges of each plant species.


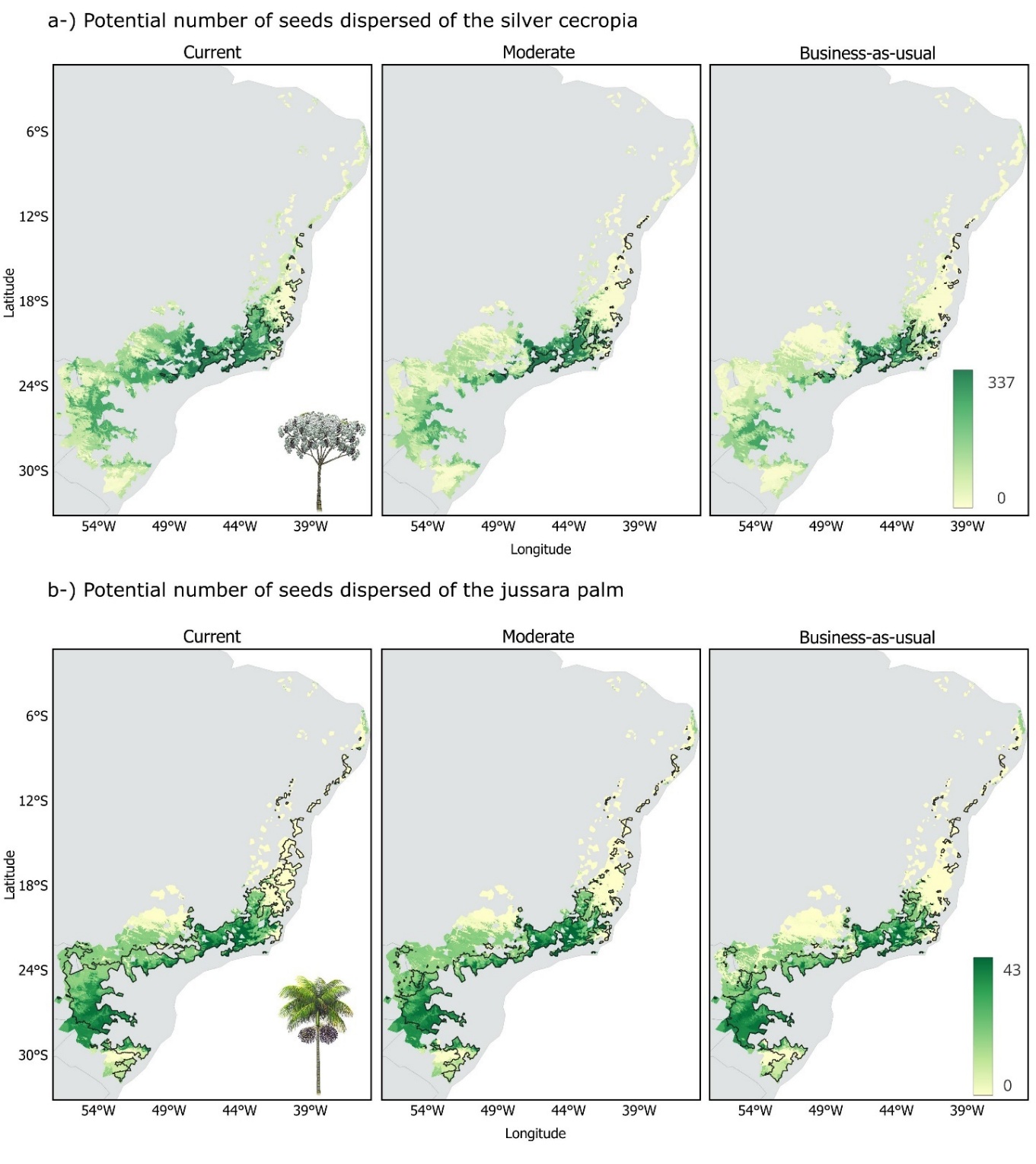


**Supplementary Fig. 2** **| Quantitative seed dispersal by frugivores of two keystone plants under current and future climates.** (**a**) Seed dispersers of silver cecropia (Cecropia hololeuca). (**b**) Seed dispersers of jussara palm (Euterpe edulis). Maps show the total number of seeds dispersed per grid cell under current conditions (left) and projected numbers under moderate (middle) and business-as-usual (right) climate scenarios. Black contour lines mark current and forecasted plant ranges


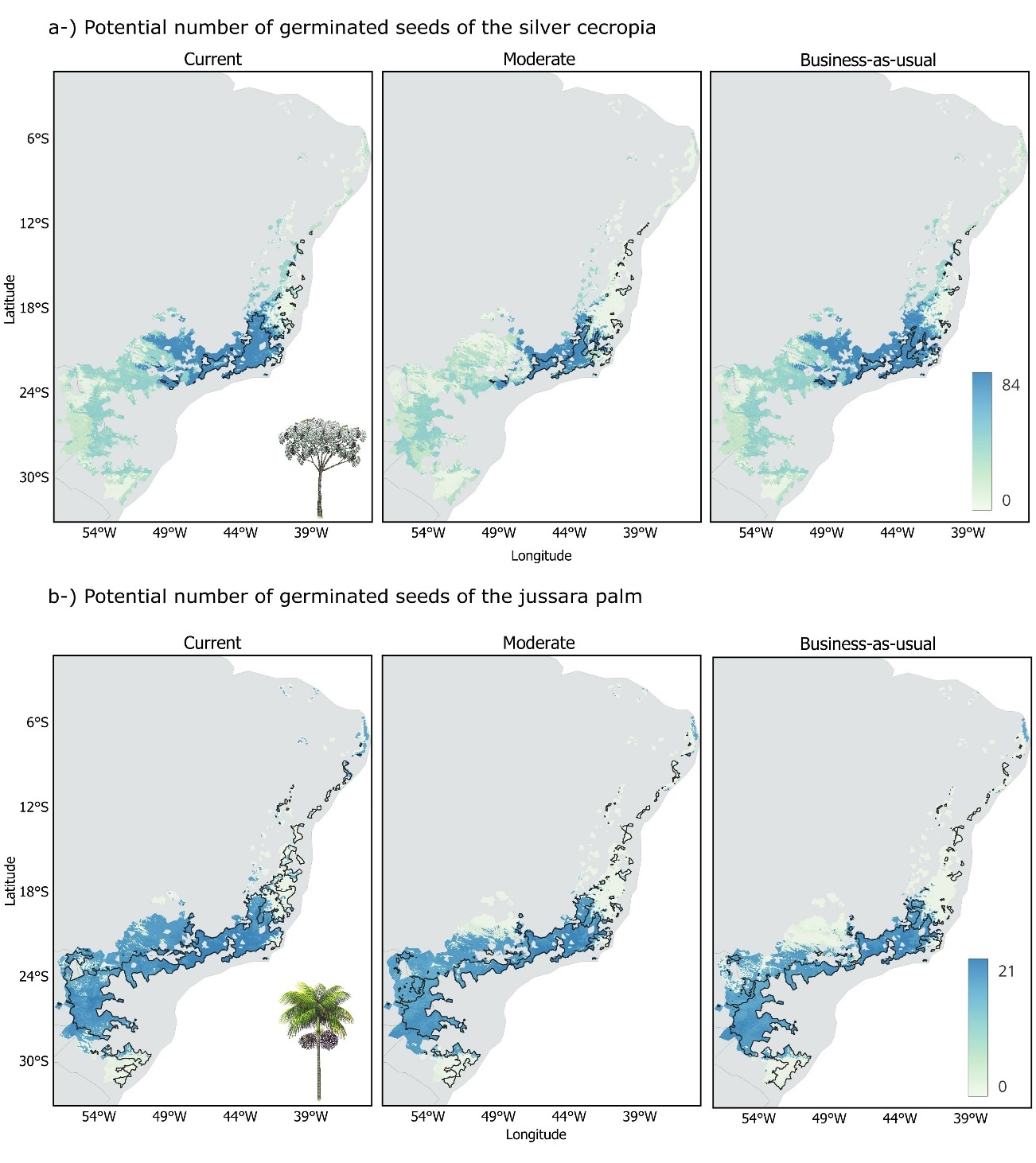


**Supplementary Fig. 3** **| Seed dispersal effectiveness of frugivores under current and future climates.** (**a**) Seed dispersers of silver cecropia (*Cecropia hololeuca*). (**b**) Seed dispersers of jussara palm (*Euterpe edulis*). Maps show the total seed dispersal effectiveness per grid cell (number of seeds likely to germinate) under current conditions (left) and projected under moderate (middle) and business-as-usual (right) climate scenarios. See Supplementary Table S3 for included frugivores. Black contour lines indicate current and forecasted plant ranges.


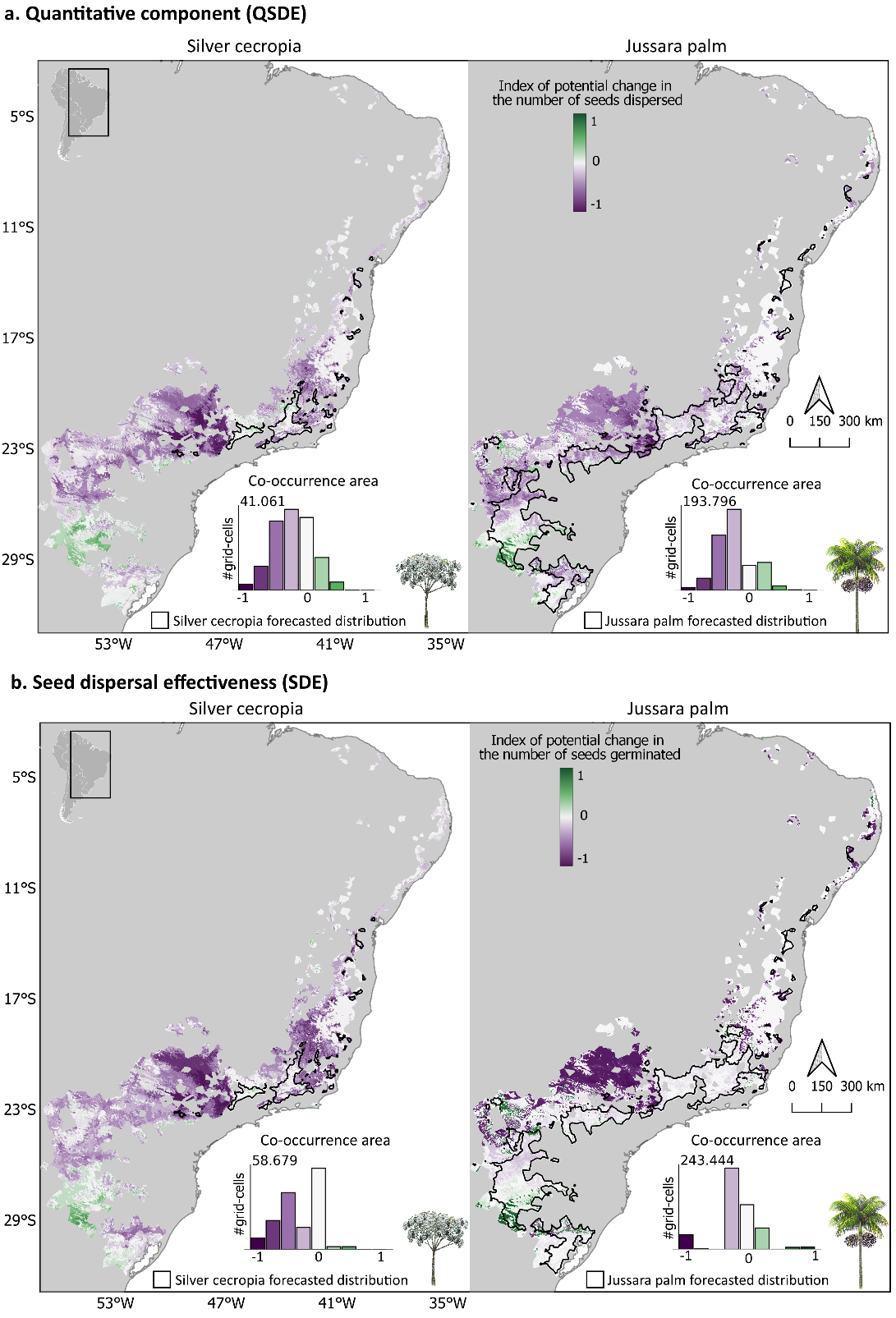


**Supplementary Fig. 4| Projected climate impacts on seed dispersal and germination.** Maps show forecasted changes under a business-as-usual climate scenario for (**a**) the quantitative component of seed dispersal (qSDE) and (**b**) total seed dispersal effectiveness (SDE) in the semideciduous Atlantic Forest. Positive values (green) indicate increases, and negative values (purple) indicate declines in the number of seeds dispersed or germinated. Results are shown separately for silver cecropia (Cecropia hololeuca, left) and jussara palm (Euterpe edulis, right), with projected plant ranges outlined in black. Insets display the frequency distribution of (q)SDE index values within each species’ forecasted co-occurrence area.


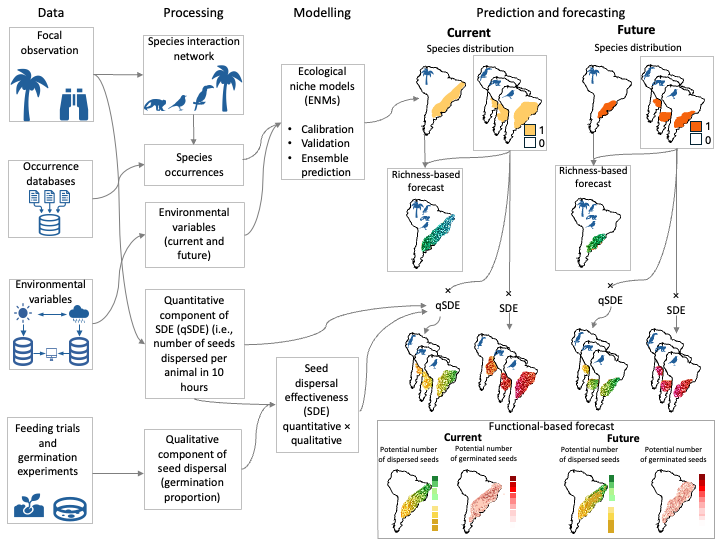


**Supplementary Fig. 5**| **Workflow of data integration, modelling, and forecasting. Data from focal** frugivore **observations, occurrence databases, environmental layers (climate, soil), and experimental feeding and germination trials were** integrated **to model species distributions and** estimate **the quantitative** and qualitative **components of seed dispersal effectiveness (SDE). Richness-based forecasts** were generated by overlaying frugivore and plant distributions to quantify potential co-occurrence and disperser richness per grid cell. Function-based forecasts combined species distributions with qSDE and SDE to estimate the number of seeds dispersed and germinated per grid cell under current and future climate scenarios.


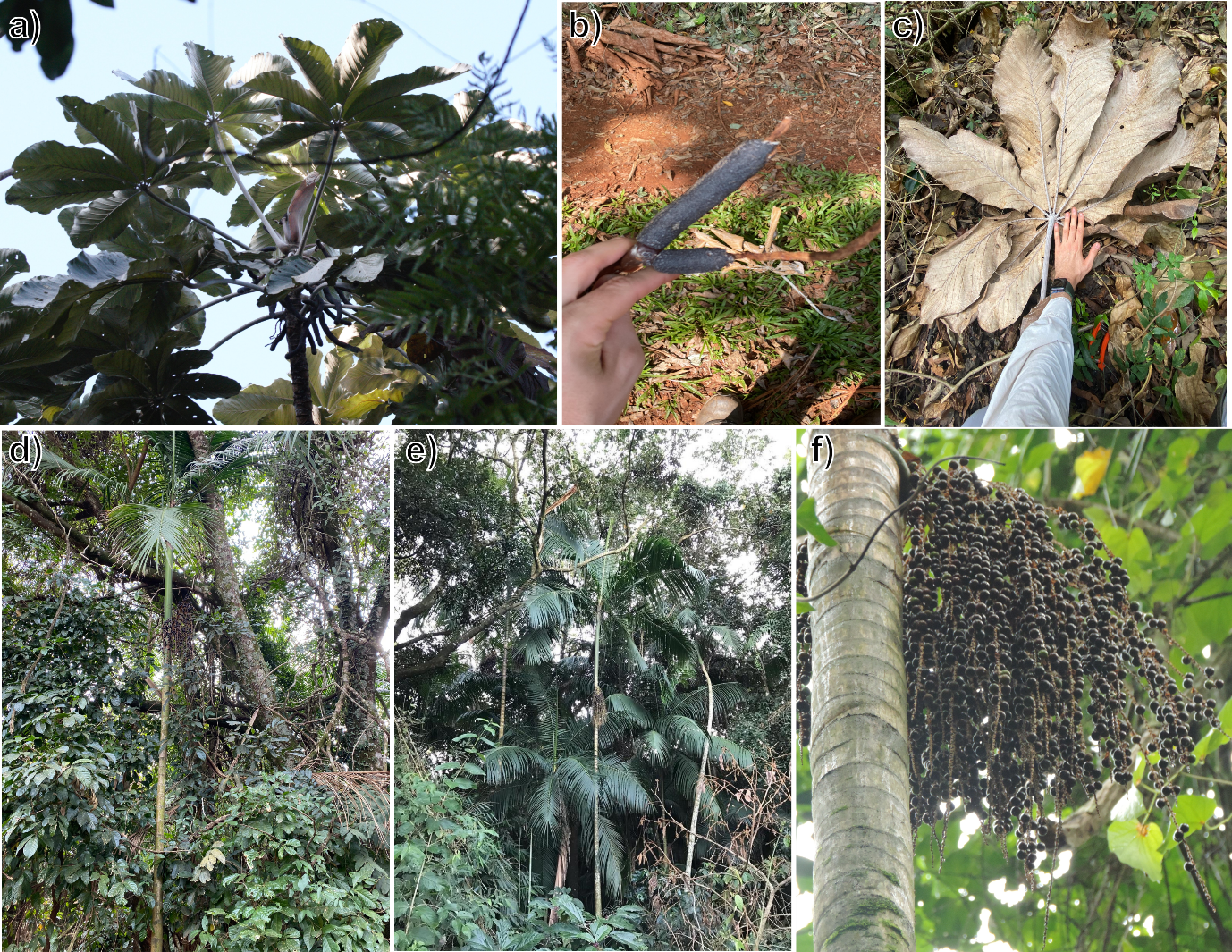


**Supplementary Fig. 6| Keystone plant species and their fruits.** (**a–c**) Silver cecropia (Cecropia hololeuca): tree with ripe infructescences at Parque Estadual da ARA in Valinhos, Brazil (**a**), infructescence with spadices pecked by birds (**b**), and characteristic leaf at ARIE Mata Santa Genebra, Brazil (**c**). (**d–f**) Jussara palm (Euterpe edulis): tree with ripe fruits at ARIE Mata Santa Genebra, Brazil (**d–e**) and at Estação Ecológica dos Caetetus, Brazil (**f**). All photographs were taken by Eduardo D. B. Rigacci during focal-tree observations in the Atlantic Forest, Brazil.


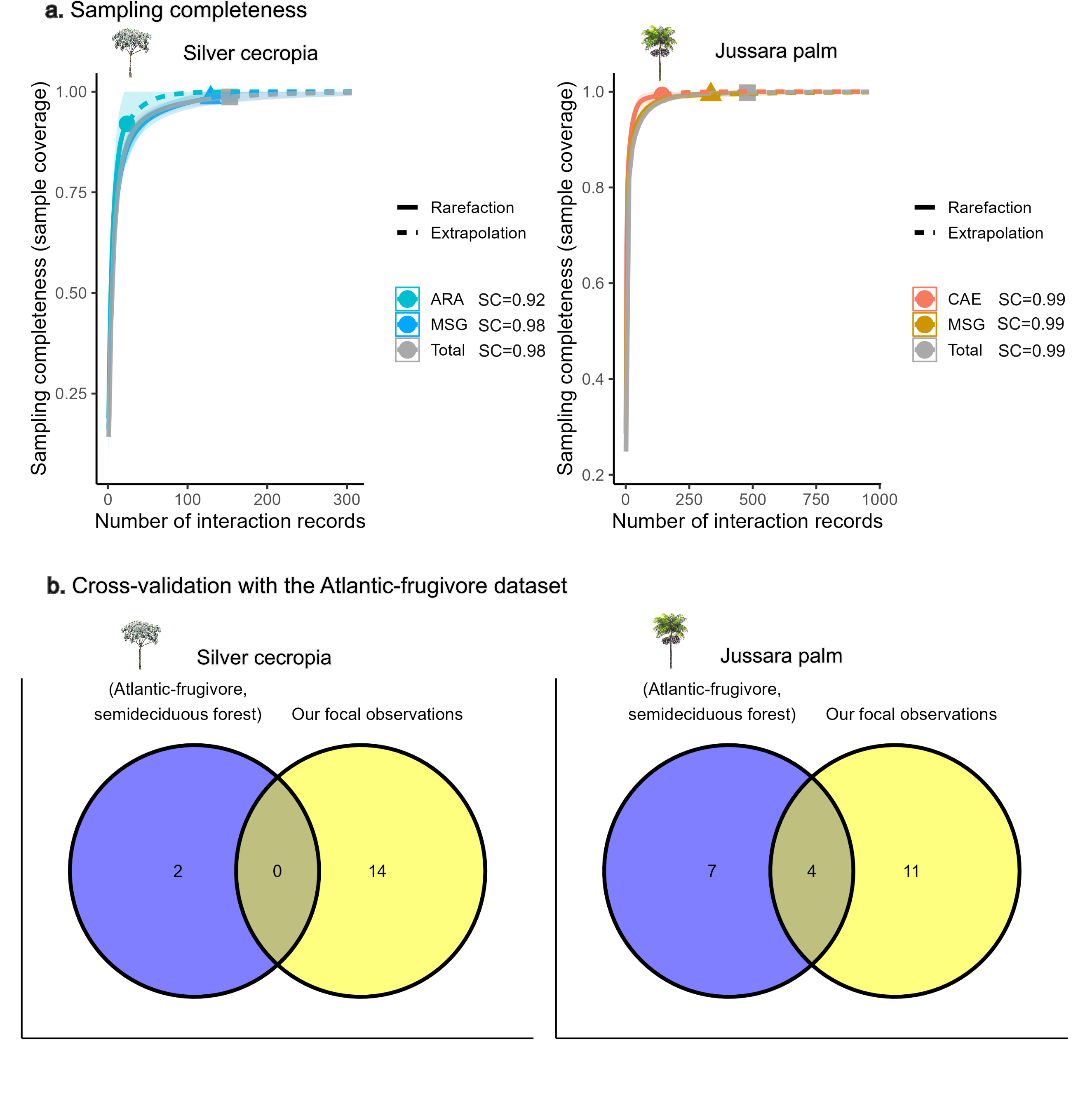


**Supplementary Fig. 7| Robustness of interaction data.** (**a**) Sample completeness (sample coverage) curves for seed dispersal interactions of silver cecropia (left) and jussara palm (right) across sampling areas: ARA (Parque Estadual do ARA), MSG (ARIE Mata Santa Genebra), and CAE (Estação Ecológica dos Caetetus). Curves were estimated using interaction records (visits resulting in seed dispersal events) as sampling units, treating each unique plant–frugivore pair as a “species” (q = 0). Solid and dashed segments show rarefaction and extrapolation, respectively; shaded bands denote 95% confidence intervals. Colored lines represent area-specific estimates, and grey lines represent pooled data across areas (“Total”). Sample completeness (SC) values indicate the observed sample coverage for each area and for pooled data. (**b**) Cross-check of the observed frugivore assemblage for each focal plant against published interaction records from the Atlantic-Frugivore database restricted to semideciduous Atlantic Forest. Venn diagrams show the number of frugivore species shared between datasets and those unique to each source.

**Supplementary Table 1 | Frugivore data for silver cecropia (*Cecropia hololeuca*).** The table summarizes species-level information on frugivore morphology (body mass, beak length, width and calculated volume), focal observations (visits per 10 h, mean number of pecks, and mean number seeds dispersed per visit), and the quantitative and qualitative components of seed dispersal. The derived seed dispersal effectiveness (SDE) is also reported. Note that the qualitative component and SDE are only available for species included in captive feeding trials. Mean body mass of bird species was obtained from the Atlantic Bird Traits^1^, while mean body mass of primates was derived from the ecological traits of the world’s primate’s dataset^2^.

| Taxonomic name | Common english name | Family | Body mass (g) | Beak length (mm) | Beak width (mm) | Beak volume (mm^3^) | Total of visits | Mean number of pecks per visit | Number of visits per 10h | Number of seeds dispersed per visit | Quantitative component | Qualitative component (mean germination proportion) | SDE |
| --- | --- | --- | --- | --- | --- | --- | --- | --- | --- | --- | --- | --- | --- |
| **Birds** |  |  |  |  |  |  |  |  |  |  |  |  |  |
| *Cyclarhis gujanensis* | Rufous-browed Peppershrike | Vireonidae | 27.4 ± 3.4 | 16.6 ± 1.1 | 11.8 ± 0.5 | 533.52 | 1 | 2 | 0.11±0.07 | 7.1 ± 0 | 0.80 | - | - |
| *Dacnis cayana* | Blue Dacnis | Thraupidae | 13.3 ± 2.2 | 14.02 ± 0.8 | 7.4 ± 1 | 201.79 | 19 | 3.68 ± 3 | 2.15±0.8 | 4.95 ± 4 | 10.69 | - | - |
| *Euphonia chlorotica* | Purple-throated Euphonia | Fringillidae | 11 ± 1.8 | 9.7 ± 0.7 | 8.09 ± 0.7 | 162.52 | 2 | 2.5 ± 2.1 | 0.22±0.1 | 2.7 ± 2.2 | 0.61 | - | - |
| *Myiodynastes solitarius* | Southern Streaked Flycatcher | Tyrannidae | 45.2 ± 4.8 | 23.67 ± 2.5 | 17.03 ± 1 | 1785.57 | 3 | 1.67 ± 0.5 | 0.34 ± 0.1 | 19.8 ± 6.8 | 6.75 | - | - |
| *Pitangus sulphuratus* | Great Kiskadee | Tyrannidae | 61.7 ± 7.6 | 27.6 ± 1.8 | 16.22 ± 1.3 | 1896.30 | 33 | 1.45 ± 1 | 3.74 ± 0.7 | 18.3 ± 12.6 | 68.88 | 0.35 ± 0.18 | 24.31 |
| *Ramphocelus carbo* | Silver-beaked Tanager | Thraupidae | 27.7 ± 2.9 | 17.4 ± 1.8 | 14.3 ± 0.4 | 931.51 | 4 | 3.25 ± 2.8 | 0.45 ± 0.2 | 20.1 ± 17.8 | 9.16 | - | - |
| *Saltator similis* | Green-winged Saltator | Thraupidae | 46.8 ± 5.4 | 19.5 ± 0.8 | 13.1 ± 0.57 | 876.08 | 2 | 6.5 ± 0.7 | 0.22 ± 0.1 | 37.9 ± 4.1 | 8.61 | 0.3 ± 0.02 | 2.64 |
| *Stilpnia cayana* | Burnished-buff Tanager | Thraupidae | 19.4 ± 2.2 | 12.7 ± 0.7 | 9.1 ± 0.8 | 275.33 | 1 | 3 | 0.11 ± 0.07 | 5.5 ± 0 | 0.62 | - | - |
| *Tachyphonus coronatus* | Ruby-crowned Tanager | Thraupidae | 27.5 ± 6 | 16.6 ± 0.3 | 10.1 ± 1.3 | 443.32 | 16 | 2.12 ± 1.3 | 1.81 ± 0.4 | 6.3 ± 4.1 | 11.40 | - | - |
| *Thraupis palmarum* | Palm Tanager | Thraupidae | 37.5 ± 4.4 | 14.4 ± 0.8 | 9.2 ± 0.8 | 320.47 | 4 | 3 ± 1 | 0.45 ± 0.1 | 6.4 ± 2.13 | 2.9 | - | - |
| *Thraupis sayaca* | Sayaca tanager | Thraupidae | 33.9 ± 4.6 | 13.8 ± 0.5 | 9.8 ± 0.9 | 346.97 | 37 | 2.78 ± 2 | 4.2 ± 0.9 | 6.49 ± 4.6 | 27.24 | 0.53 ± 0.02 | 14.48 |
| *Turdus leucomelas* | Pale-breasted Thrush | Turdidae | 63.4 ± 8.38 | 19.3 ± 0.98 | 10.7 ± 1.3 | 578.48 | 11 | 3.27 ± 2.5 | 1.24 ± 0.3 | 12.9 ± 10 | 16.16 | 0.193 ± 0.03 | 3.12 |
| **Primates** |  |  |  |  |  |  |  |  |  |  |  |  |  |
| *Callithrix penicillata* | Black-pencilled Marmoset | Callitrichidae | 330 | 15.3 ± 0.91 | - | - | 7 | 5.5 ± 3.3 | 0.79 ± 0.2 | 94.7 ± 56.2 | 59.67 | 0.67 ± 0.4 | 39.78 |
| *Sapajus nigritus* | Black Capuchin | Cebidae | 3700 | 29.89 ± 1.2 | - | - | 13 | 1.53 ± 0.6 | 2.27 ± 0.3 | 50.02 ± 21.9 | 113.58 | - | - |

**Supplementary Table 2 | Frugivore data for jussara palm (*Euterpe edulis*).** The table summarizes species-level information on frugivore focal observations (visits per 10 h and mean seeds dispersed per visit), the quantitative and qualitative components of seed dispersal, and the derived seed dispersal effectiveness (SDE). The qualitative component and SDE are reported only for species included in captive feeding trials. Mean body mass of bird species was obtained from the Atlantic Bird Traits^1^, while mean body mass of primates was derived from the ecological traits of the world’s primates dataset^2^.

| Taxonomic name | Common english name | Family | Body mass (g) | Total of visits | No. visits/10h | No. of seeds per visit | Quantitative component | Qualitative component (mean germination proportion) | SDE |
| --- | --- | --- | --- | --- | --- | --- | --- | --- | --- |
| **Birds** |  |  |  |  |  |  |  |  |  |
| *Baryphthengus ruficapillus* | Rufous-capped Motmot | Momotidae | 130.8 ± 22.5 | 8 | 0.3874 ± 0.15 | 2.75 ± 1.8 | 1.06 |  |  |
| *Megarynchus pitangua* | Boat-billed Flycatcher | Tyrannidae | 57.2 ± 7.9 | 2 | 0.0968 ± 0.03 | 1 ± 0 | 0.09 |  |  |
| *Pitangus sulphuratus* | Great Kiskadee | Tyrannidae | 61.7 ± 7.6 | 4 | 0.1937 ± 0.01 | 1 ± 0 | 0.19 |  |  |
| *Penelope superciliaris* | Rusty-margined Guan | Cracidae | 741 ± 221 | 8 | 0.3874 ± 0.07 | 2 ± 1 | 0.77 | 0.910±0.1 | 0.71 |
| *Ramphastos toco* | Toco Toucan | Ramphastidae | 650 ± 84.8 | 5 | 0.2421 ± 0.07 | 6.8 ± 2.7 | 1.64 | 0.9±0.07 | 1.49 |
| *Saltator similis* | Green-winged saltator | Thraupidae | 46.8 ± 5.4 | 5 | 0.2421 ± 0.13 | 1.4 ± 0.5 | 0.33 | 0.37±0.2 | 0.12 |
| *Tachyphonus coronatus* | Ruby-crowned Tanager | Thraupidae | 27.5 ± 6 | 1 | 0.0484 ± 0.03 | 1 | 0.04 |  |  |
| *Turdus albicollis* | White-necked Thrush | Turdidae | 66.7 ± 7.7 | 84 | 4.0677 ± 0.9 | 1.69 ± 0.8 | 6.87 |  |  |
| *Turdus amaurochalinus* | Creamy-bellied Thrush | Turdidae | 59.6 ± 7.6 | 93 | 4.5036 ± 0.6 | 1.74 ± 0.9 | 7.84 |  |  |
| *Turdus flavipes* | Yellow-legged Thrush | Turdidae | 60.3 ± 7.3 | 7 | 0.3389 ± 0.1 | 1.42 ± 0.5 | 0.48 |  |  |
| *Turdus leucomelas* | Pale-breasted Thrush | Turdidae | 64.4 ± 8.38 | 201 | 9.7336 ± 1 | 1.66 ± 0.9 | 16.17 | 1 | 16.17 |
| *Turdus rufiventris* | Rufous-bellied Thrush | Turdidae | 72.4 ± 7.3 | 21 | 1.0169 ± 0.25 | 1.9 ± 0.9 | 1.93 | 0.71±0.25 | 1.38 |
| *Turdus subalaris* | Eastern Slaty Thrush | Turdidae | 54 ± 7.3 | 17 | 0.8232 ± 0.2 | 2 ± 0.9 | 1.64 |  |  |
| *Thraupis palmarum* | Palm Tanager | Thraupidae | 37.5 ± 4.4 | 6 | 0.0484 ± 0.15 | 1 ± 0 | 0.19 |  |  |
| **Primate** |  |  |  |  |  |  |  |  |  |
| *Sapajus nigritus* | Black-horned Capuchin | Cebidae | 3700 | 17 | 0.8232 ± 0.21 | 4.29 ± 2.7 | 3.53 | 0.33±0.1 | 1.16 |

**Supplementary Table 3 | Predicted range sizes and changes under climate change.** The table reports species’ geographic range sizes (km^2^) and projected range changes (%) in the semideciduous forest under moderate and business-as-usual (B.A.U.) climate scenarios. Percentages indicate relative expansion or contraction compared with current distributions.

|  |  |  | Current |  | Climate change | | | | |
| --- | --- | --- | --- | --- | --- | --- | --- | --- | --- |
|  |  |  |  |  | Moderate | |  | B.A.U | |
| Taxonomic name | Common english name |  | km^2^ |  | Range change (%) | km^2^ |  | Range change (%) | km^2^ |
| **Plants** |  |  |  |  |  |  |  |  |  |
| *Cecropia hololeuca* | Silver cecropia |  | 164,644.2 |  | -20.6 | 130,635.7 |  | -34.3 | 108,090.8 |
| *Euterpe edulis* | Jussara palm |  | 512,801 |  | -14.3 | 439,267.5 |  | -24.3 | 387,876 |
| **Birds** |  |  |  |  |  |  |  |  |  |
| *Cyclarhis gujanensis* | Rufous-browed Peppershrike |  | 363,411 |  | -38.5 | 223,328 |  | -50.3 | 180,316 |
| *Dacnis cayana* | Blue Dacnis |  | 424,155 |  | -0.3 | 422,757 |  | -25.0 | 317,787 |
| *Euphonia chlorotica* | Purple-throated Euphonia |  | 548,976 |  | -8.0 | 504,966 |  | -13.6 | 474,112 |
| *Myiodynastes solitarius* | Southern Streaked Flycatcher |  | 451,793 |  | -15.7 | 380,810 |  | -29.7 | 317,517 |
| *Ramphocelus carbo* | Silver-beaked Tanager |  | 174,612 |  | 35.0 | 235,806 |  | -33.4 | 116,141 |
| *Stilpnia cayana* | Burnished-buff Tanager |  | 279,041 |  | -49.5 | 140,748 |  | -56.3 | 121,784 |
| *Saltator similis* | Green-winged Saltator |  | 352,965 |  | -44.0 | 197,606 |  | -38.7 | 216,359 |
| *Tachyphonus coronatus* | Ruby-crowned Tanager |  | 445,242 |  | -34.6 | 290,974 |  | -41.9 | 258,297 |
| *Turdus leucomelas* | Pale-breasted Thrush |  | 600,677 |  | -10.7 | 536,249 |  | -23.2 | 461,094 |
| *Thraupis sayaca* | Sayaca Tanager |  | 505,440 |  | -32.1 | 343,138 |  | -68.4 | 159,304 |
| *Baryphthengus ruficapillus* | Rufous-capped Motmot |  | 465,070 |  | -12.2 | 407,971 |  | -18.2 | 380,058 |
| *Megarynchus pitangua* | Boat-billed Flycatcher |  | 412,473 |  | 7.2 | 442,387 |  | -2.7 | 401,200 |
| *Pitangus sulphuratus* | Great Kiskadee |  | 521,093 |  | -24.4 | 393,665 |  | -38.7 | 319,412 |
| *Penelope superciliaris* | Rusty-margined Guan |  | 551,368 |  | -7.9 | 507,267 |  | -21.7 | 431,335 |
| *Ramphastos toco* | Toco Toucan |  | 378,609 |  | 0.5 | 380,591 |  | -6.8 | 352,837 |
| *Turdus albicollis* | White-necked Thrush |  | 301,898 |  | -25.2 | 225,568 |  | -33.5 | 200,591 |
| *Turdus amaurochalinus* | Creamy-bellied Thrush |  | 378,960 |  | -39.6 | 228,753 |  | -55.3 | 169,348 |
| *Turdus flavipes* | Yellow-legged Thrush |  | 208,184 |  | -33.4 | 138,508 |  | -46.4 | 111,471 |
| *Turdus rufiventris* | Rufous-bellied Thrush |  | 313,738 |  | -43.7 | 176,627 |  | -58.2 | 131,100 |
| *Turdus subalaris* | Eastern Slaty Thrush |  | 347,714 |  | -45.8 | 188,218 |  | -53.2 | 162,547 |
| *Thraupis palmarum* | Palm Tanager |  | 261,752 |  | -21.9 | 204,288 |  | -44.7 | 144,553 |
| **Primates** |  |  |  |  |  |  |  |  |  |
| *Callithrix penicillata* | Black-pencilled Marmoset |  | 295,619 |  | -44.2 | 164,765 |  | -49.8 | 148,246 |
| *Sapajus nigritus* | Black-horned Capuchin |  | 263,858 |  | -43.2 | 149,688 |  | -49.2 | 133,918 |

**Supplementary Table 4 | Predicted distributions and co-occurrence with seed dispersers.** The table reports the current and projected geographic ranges (km²) and co-occurrence (%) of the silver cecropia (Cecropia hololeuca) or jussara palm (Euterpe edulis) and their associated frugivore communities under moderate and business-as-usual (B.A.U.) climate scenarios. “Interaction” indicates the plant species which seed dispersers interact with (CH = Cecropia hololeuca; EE = Euterpe edulis), while "iniDist" and "forDist" denote current and future geographic range sizes (km^2^). “Co-occurrence" shows the percentage of each plant’s suitable area occupied by a disperser.

|  |  |  |  | Current | |  | Climate change | | | | | | |
| --- | --- | --- | --- | --- | --- | --- | --- | --- | --- | --- | --- | --- | --- |
|  |  |  |  |  | | | Moderate | | |  | B.A.U | | |
| Species |  | Interaction |  | iniDist (km^2^) | Co-occurrence (%) |  | forDist (km^2^) | Co-occurrence (%) | Change in co-occurence (%) |  | forDist (km^2^) | Co-occurrence (%) | Change in co-occurence (%) |
| ***Cecropia hololeuca*** |  |  |  | 164,644.2 |  |  | 130,635.7 |  |  |  | 108,090.8 |  |  |
| *Cyclarhis gujanensis* |  | CH |  | 100,243.9 | 60.8 |  | 69,034.8 | 52.8 | -31.1 |  | 54,448.6 | 50.3 | -45.6 |
| *Callithrix penicillata* |  | CH |  | 133,564.5 | 81.1 |  | 82,923.9 | 63.4 | -37.9 |  | 65,988.3 | 61.0 | -50.5 |
| *Dacnis cayana* |  | CH |  | 129,834.2 | 78.8 |  | 95,622.3 | 73.1 | -26.3 |  | 76,405.2 | 70.6 | -41.1 |
| *Euphonia chlorotica* |  | CH |  | 125,570.8 | 76.2 |  | 97,515.4 | 74.6 | -22.3 |  | 81,063.2 | 74.9 | -35.4 |
| *Myiodynastes solitarius* |  | CH |  | 104,748.9 | 63.6 |  | 79,738.7 | 61.0 | -23.8 |  | 66,647.9 | 61.6 | -36.3 |
| *Pitangus sulphuratus* |  | CH |  | 131,405 | 79.8 |  | 94,120.1 | 72.0 | -28.3 |  | 68,422 | 63.3 | -47.9 |
| *Ramphocelus carbo* |  | CH |  | 34,226.29 | 20.7 |  | 44,480.3 | 34.0 | 29.9 |  | 33,675.1 | 31.1 | -1.6 |
| *Stilpnia cayana* |  | CH |  | 122,744.3 | 74.5 |  | 82,334.9 | 63.0 | -32.9 |  | 64,734.1 | 59.8 | -47.2 |
| *Sapajus nigritus* |  | CH |  | 79,296.76 | 48.1 |  | 63,461.3 | 48.5 | -19.9 |  | 51,980.7 | 48.0 | -34.4 |
| *Saltator similis* |  | CH |  | 100,427.7 | 60.9 |  | 59,961.1 | 45.8 | -40.2 |  | 53,460.8 | 49.4 | -46.7 |
| *Tachyphonus coronatus* |  | CH |  | 108,683 | 66.0 |  | 73,357.8 | 56.1 | -32.5 |  | 57,571.4 | 53.2 | -47.0 |
| *Turdus leucomelas* |  | CH |  | 128,719.5 | 78.1 |  | 96,696.6 | 74.0 | -24.8 |  | 77,909.3 | 72.0 | -39.4 |
| *Thraupis palmarum* |  | CH |  | 122,070.5 | 74.1 |  | 87,481.2 | 66.9 | -28.3 |  | 65,305.2 | 60.4 | -46.5 |
| *Thraupis sayaca* |  | CH |  | 128,866.4 | 78.2 |  | 85,432.3 | 65.3 | -33.7 |  | 46,598.1 | 43.1 | -63.8 |
|  |  |  |  |  |  |  |  |  |  |  |  |  |  |
| ***Euterpe edulis*** |  |  |  | 512,801 |  |  | 43,9267.5 |  |  |  | 382,367 |  |  |
| *Baryphthengus ruficapillus* |  | EE |  | 364,653 | 71.1 |  | 299,028 | 68.0 | -17.9 |  | 250,985 | 65.6 | -31.1 |
| *Megarynchus pitangua* |  | EE |  | 290,452 | 56.6 |  | 287,920 | 65.5 | -0.8 |  | 254,937 | 66.6 | -12.2 |
| *Pitangus sulphuratus* |  | EE |  | 350,839 | 68.4 |  | 271,695 | 61.8 | -22.5 |  | 211,573 | 55.3 | -39.6 |
| *Penelope superciliaris* |  | EE |  | 361,311 | 70.4 |  | 307,370 | 69.9 | -14.9 |  | 211,573 | 55.3 | -26.5 |
| *Ramphastos toco* |  | EE |  | 223,103 | 43.5 |  | 200,133 | 45.5 | -10.2 |  | 184,263 | 48.1 | -17.4 |
| *Sapajus nigritus* |  | EE |  | 236,012 | 46.0 |  | 148,397 | 33.7 | -37.1 |  | 131,600 | 34.4 | -44.2 |
| *Saltator similis* |  | EE |  | 287,914 | 56.1 |  | 170,816 | 38.8 | -40.6 |  | 168,756 | 44.1 | -41.3 |
| *Tachyphonus coronatus* |  | EE |  | 373,544 | 72.8 |  | 257,686 | 58.6 | -31.0 |  | 209,103 | 54.6 | -44.0 |
| *Turdus albicollis* |  | EE |  | 237,367 | 46.2 |  | 183,120 | 41.6 | -22.8 |  | 147,102 | 38.4 | -38.0 |
| *Turdus amaurochalinus* |  | EE |  | 294580 | 57.4 |  | 188,427 | 42.8 | -36.0 |  | 124,490 | 32.5 | -57.7 |
| *Turdus flavipes* |  | EE |  | 196,870 | 38.3 |  | 125,888 | 28.6 | -36.0 |  | 103,041 | 26.9 | -47.6 |
| *Turdus leucomelas* |  | EE |  | 393,894 | 76.8 |  | 335,835 | 76.4 | -14.7 |  | 287,239 | 75.1 | -27.0 |
| *Turdus rufiventris* |  | EE |  | 250,394 | 48.8 |  | 141,764 | 32.2 | -43.3 |  | 88,683 | 23.1 | -64.5 |
| *Turdus subalaris* |  | EE |  | 291,842 | 56.9 |  | 165,860 | 37.7 | -43.1 |  | 125,279 | 32.7 | -57.0 |
| *Thraupis palmarum* |  | EE |  | 195,338 | 38.0 |  | 131,934 | 30.0 | -32.4 |  | 97,692.2 | 25.5 | -49.9 |

**Supplementary Table 5 | Forecasted changes in frugivore contributions to the silver cecropia (Cecropia hololeuca)**. The table shows the predicted contribution of individual frugivores to the quantitative component of seed dispersal and seed dispersal effectiveness for Cecropia hololeuca. 'Current' indicates the number of seeds dispersed or germinated across all grid cells under present conditions. Values under moderate and a business-as-usual (B.A.U.) scenarios indicate projected numbers and the percentage change relative to the current predictions.

|  |  | Quantitative component of seed dispersal (potential number of dispersed seeds) | | | | |  | | Seed dispersal effectiveness (potential number of germinated seeds) | | | | |  |
| --- | --- | --- | --- | --- | --- | --- | --- | --- | --- | --- | --- | --- | --- | --- |
|  | Current | Climate change scenario | | | | | Current | | Climate change scenario | | | | |  |
|  |  | Moderate | | B.A.U. | | |  |  | Moderate | |  | B.A.U. | |  |
| Taxonomic name | # seeds | % | # seeds |  | % | # seeds |  | # seeds | % | # seeds |  | % | # seeds | |
| **Birds** |  |  |  |  |  |  |  |  |  |  |  |  |  | |
| *Cyclarhis gujanensis* | 100,472.8 | -30.9 | 69,378.4 |  | -45.5 | 54,750.4 |  |  |  |  |  |  |  | |
| *Dacnis cayana* | 1,735,564.26 | -26.2 | 1,281,506.51 |  | -40.9 | 1,025,929.99 |  |  |  |  |  |  |  | |
| *Euphonia chlorotica* | 95,830.39 | -22.2 | 74,568.84 |  | -35.2 | 62,110.2 |  |  |  |  |  |  |  | |
| *Myiodynastes solitarius* | 88,6470.75 | -23.8 | 675,681.75 |  | -33.2 | 592,016.12 |  |  |  |  |  |  |  | |
| *Pitangus sulphuratus* | 11,318,154.96 | -28.2 | 8,126,530.96 |  | -47.7 | 5,920,167.12 |  | 3,994,546.27 | -28.2 | 2,868,118.11 |  | -38.8 | 2,444,953.9 | |
| *Ramphocelus carbo* | 393,302.92 | 30.2 | 512,126.44 |  | -1.2 | 388,777.88 |  |  |  |  |  |  |  | |
| *Stilpnia cayana* | 95,142.72 | -32.7 | 63,988.34 |  | -47.0 | 50,381.82 |  |  |  |  |  |  |  | |
| *Saltator similis* | 1,083,999 | -40.2 | 647,945.55 |  | -46.6 | 578,333.7 |  | 332,376 | -40.2 | 198,673.2 |  | -47.0 | 176,278.08 | |
| *Tachyphonus coronatus* | 1,553,170.15 | -32.4 | 1,049,620.76 |  | -46.9 | 824,995.17 |  |  |  |  |  |  |  | |
| *Turdus leucomelas* | 2,602,260.94 | -24.7 | 1,958,769.74 |  | -39.2 | 1,581,611.51 |  | 842,094.21 | -1.5 | 829,336.53 |  | -2.2 | 823,935.81 | |
| *Thraupis palmarum* | 442,661.8 | -28.1 | 318,069.1 |  | -46.3 | 237,776.8 |  |  |  |  |  |  |  | |
| *Thraupis sayaca* | 4,390,733.88 | -33.5 | 2,919,937.32 |  | -63.7 | 1,595,596.31 |  | 2,333,987.76 | -33.5 | 1,552,154.59 |  | -42.1 | 1,350,665.4 | |
| **Primates** |  |  |  |  |  |  |  |  |  |  |  |  |  | |
| *Callithrix penicillata* | 9,954,865.44 | -37.7 | 6,199,474.32 |  | -50.4 | 4,937,036.13 |  | 6,636,576.96 | -37.7 | 4,132,982.88 |  | -47.8 | 3,466,270.1 | |
| *Sapajus nigritus* | 11,316,997.62 | -20.0 | 9,056,869.2 |  | -34.4 | 7,425,973.98 |  |  |  |  |  |  |  | |
|  |  |  |  |  |  |  |  |  |  |  |  |  |  | |
| Total of dispersed seeds | 45,969,627.63 | -28.31 | 32,954,467.23 |  | -45.01 | 25,275,457.13 | Total of germinated seeds | 14,139,581.2 | -32.23 | 9,581,265.31 |  | -41.56 | 8,262,103.31 | |

**Supplementary Table 6 | Forecasted changes in frugivores contributions to the jussara palm (Euterpe edulis)**. The table shows predicted contributions of individual frugivores to the quantitative component of seed dispersal and seed dispersal effectiveness for Euterpe edulis. 'Current' indicates the number of seeds dispersed or germinated across all grid cells under present conditions. Values under moderate and a business-as-usual (B.A.U.) scenarios show projected numbers and the change relative to the current predictions.

|  | |  | Quantitative component of seed dispersal (potential number of dispersed seeds) | | | | |  | | Seed dispersal effectiveness (potential number of germinated seeds) | | | | | | |  |
| --- | --- | --- | --- | --- | --- | --- | --- | --- | --- | --- | --- | --- | --- | --- | --- | --- | --- |
|  | | Current | Climate change scenario | | | | | Current | | Climate change scenario | | | | | | |  |
|  | |  | Moderate | | B.A.U. | | |  |  | Moderate | |  | | B.A.U. | | |  |
| Taxonomic name | | # seeds | % | # seeds |  | % | # seeds |  | # seeds | % | # seeds | |  | | % | # seeds | |
| **Birds** | |  |  |  |  |  |  |  |  |  |  | |  | |  |  | |
| *Baryphthengus ruficapillus* | | 491,732 | -17.7 | 404,897 |  | -30.80 | 340,144 |  |  |  |  | |  | |  |  | |
| *Megarynchus pitangua* | | 33,053 | -0.4 | 32,906 |  | -3.10 | 32,029 |  |  |  |  | |  | |  |  | |
| *Pitangus sulphuratus* | | 84,599 | -22.2 | 65,785 |  | -39.20 | 51,404 |  |  |  |  | |  | |  |  | |
| *Penelope superciliaris* | | 352,222 | -14.7 | 300,477 |  | -26.30 | 259,550 |  | 324,776 | -14.7 | 277,063 | |  | | -26.3 | 239,325 | |
| *Ramphastos toco* | | 461,409 | -10.4 | 413,478 |  | -17.50 | 380,645 |  | 419,207 | -10.4 | 375,660 | |  | | -17.5 | 345,830 | |
| *Saltator similis* | | 180,411 | -24.8 | 135,655 |  | -29.40 | 127,393 |  | 58,511 | -14.3 | 50,165 | |  | | -19.8 | 46,941 | |
| *Tachyphonus coronatus* | | 19,102 | -30.5 | 13,268.68 |  | -43.60 | 107,74 |  |  |  |  | |  | |  |  | |
| *Turdus albicollis* | | 2,102,474 | -22.3 | 1,633,624 |  | -37.60 | 1,311,538 |  |  |  |  | |  | |  |  | |
| *Turdus amaurochalinus* | | 2,950,725 | -35.8 | 1,893,242 |  | -57.70 | 1,248,912 |  |  |  |  | |  | |  |  | |
| *Turdus flavipes* | | 119,430 | -35.5 | 76,989 |  | -46.90 | 63,386 |  |  |  |  | |  | |  |  | |
| *Turdus leucomelas* | | 8,086,148 | -14.4 | 6,921,391 |  | -26.70 | 5,920,969 |  | 8,086,148 | -14.4 | 6,921,391 | |  | | -26.8 | 5,920,969 | |
| *Turdus rufiventris* | | 619,173 | -43 | 353,051 |  | -64.30 | 221,025 |  | 442,724 | -42.9 | 252,440 | |  | | -64.3 | 158,039 | |
| *Turdus subalaris* | | 614,134 | -42.6 | 352,293 |  | -56.70 | 265,794 |  |  |  |  | |  | |  |  | |
| *Thraupis palmarum* | | 46,197 | -32.3 | 31,279 |  | -49.80 | 23,180 |  |  |  |  | |  | |  |  | |
| **Primate** | |  |  |  |  |  |  |  |  |  |  | |  | |  |  | |
| *Sapajus nigritus* | 1,060,299 | -36.9 | 669,548 |  | -44.00 | 593,940 |  | 348,426 | -37 | 219,648 | |  | | -44 | 195,175 | |  |
|  | |  |  |  |  |  |  |  |  |  |  | |  | |  |  | |
| Total of dispersed seeds | | 17,221,108 | -22.78 | 13,297,884 |  | -36.99 | 10,850,683 | Total of  germinated seeds | 9,679,792 | -16.35 | 8,096,367 | |  | | -28.65 | 6,906,279 | |

**Supplementary Table 7 |** **Decoupling between frugivore richness and seed dispersal function**. The table shows the percentage of area of each plant species’ distribution where richness underestimates function (Function > Richness), matches function (Function = Richness), and or overestimates function (Function < Richness). Results are provided for two seed dispersal metrics (qSDE and SDE) across current and future climate scenarios (Moderate and Business-as-usual, = B.A.U.). The total number of grid cells analyzed is reported in the “Total n_cells” column.

| Plant species | Decoupling index | Scenario | % Function > Richness | % Function = Richness | %Function < Richness | Total N_cells |
| --- | --- | --- | --- | --- | --- | --- |
| *Cecropia hololeuca* | qSDE – Richness | Current | 45 | 21.5 | 33.4 | 219,633 |
|  |  | Moderate | 32.2 | 36.6 | 31.07 | 177,215 |
|  |  | B.A.U. | 28.4 | 37 | 34.5 | 149,356 |
|  | SDE – Richness | Current | 21.8 | 65.49 | 12.6 | 219,633 |
|  |  | Moderate | 16.9 | 63.18 | 19.8 | 177,215 |
|  |  | B.A.U. | 10.37 | 57.10 | 32.51 | 149,356 |
| *Euterpe edulis* | qSDE - Richness | Current | 52.14 | 20.83 | 27.01 | 663,727 |
|  |  | Moderate | 60.2 | 18.3 | 21.3 | 572,649 |
|  |  | B.A.U. | 59.7 | 17.9 | 22.3 | 499,952 |
|  | SDE - Richness | Current | 57.8 | 32.9 | 9.2 | 663,727 |
|  |  | Moderate | 65 | 26.3 | 8.6 | 572,649 |
|  |  | B.A.U. | 65.4 | 26.1 | 8.3 | 499,952 |

**Supplementary Table S8 | Linear model results linking frugivore richness loss to functional loss.** Coefficient estimates from models testing the relationship between percentage loss of frugivore richness and loss of functional metrics (qSDE or SDE) under two climate scenarios (Moderate and Business-as-usual, B.A.U). Results are shown separately for silver cecropia (*Cecropia hololeuca*) and jussara palm (*Euterpe edulis*), with changes (%Δ) calculated as differences between current and future values per grid cell.

| Plant species | Scenario | Model | n_cells | Intercept | Slope | CI | R^2^ | p |
| --- | --- | --- | --- | --- | --- | --- | --- | --- |
| Jussara palm | Moderate | %ΔqSDE ~ %ΔRichness | 490,630 | -20.65 | 0.032 | [0.026, 0.037] | 0.045 | <0.001 |
|  |  | %ΔSDE ~ %ΔRichness | 466,869 | -17.00 | 0.022 | [0.022, 0.023] | 0.115 | <0.001 |
|  | B.A.U | %ΔqSDE ~ %ΔRichness | 444,668 | -27.24 | 0.104 | [0.096, 0.112] | 0.125 | <0.001 |
|  |  | %ΔSDE ~ %ΔRichness | 423,404 | -21.75 | 0.016 | [0.015, 0.016] | 0.059 | <0.001 |
| Silver cecropia | Moderate | %ΔqSDE ~ %ΔRichness | 144,598 | -20.02 | 0.037 | [0.03, 0.044] | 0.056 | <0.001 |
|  |  | %ΔSDE~ %ΔRichness | 141,605 | -1.60 | 0.906 | [0.897, 0.916] | 0.947 | <0.001 |
|  | B.A.U | %ΔqSDE ~ %ΔRichness | 120,632 | -11.08 | 0.603 | [0.546, 0.66] | 0.62 | <0.001 |
|  |  | %ΔSDE ~ %ΔRichness | 118,005 | -0.41 | 0.934 | [0.929, 0.939] | 0.964 | <0.001 |

**Supplementary Table 9** **| Occurrence records removed during data cleaning and thinning.** The 'Initial' column lists the number of occurrences compiled from all sources (see Methods in main text). Subsequent columns show the number of records remaining (# occ) and the percentage removed at each cleaning and thinning step. The 'Final' column reports the number of records retained for species distribution modelling and the total percentage reduction relative to the initial dataset.

|  | **Initial** |  | **‘Coordinate Cleaner’** | |  | **Outside range map** | |  | **Uncertainty >=1 km** | |  | **Recorded>=1979** | |  | **Thinning** | |  | **Final** | |
| --- | --- | --- | --- | --- | --- | --- | --- | --- | --- | --- | --- | --- | --- | --- | --- | --- | --- | --- | --- |
| **Taxonomic names** | **# occ** |  | **%** | **# occ** |  | **%** | **# occ** |  | **%** | **# occ** |  | **%** | **# occ** |  | **%** | **# occ** |  | **%** | **# occ** |
| *Baryphthengus ruficapillus* | 12,255 |  | -76.6 | 2,864 |  | -4.9 | 2,724 |  | -5.0 | 2,588 |  | -2.4 | 2,527 |  | -38.7 | 1,550 |  | -87.4 | 1,550 |
| *Callithrix penicillata* | 3,354 |  | -61.3 | 1,299 |  | -15.3 | 1,100 |  | -7.4 | 1,019 |  | -15.5 | 861 |  | -41.3 | 505 |  | -84.9 | 505 |
| *Cecropia hololeuca* | 361 |  | -59.0 | 148 |  | 0.0 | 148 |  | -0.7 | 147 |  | 0.0 | 147 |  | -25.9 | 109 |  | -69.8 | 109 |
| *Cyclarhis gujanensis* | 246,891 |  | -78.6 | 52,784 |  | -0.1 | 52,710 |  | -0.8 | 52,299 |  | -1.7 | 51434 |  | -44.3 | 28,661 |  | -88.4 | 28,661 |
| *Dacnis cayana* | 125,306 |  | -77.8 | 27,837 |  | 0.0 | 27,831 |  | 5.7 | 29,417 |  | -7.9 | 27,091 |  | -46 | 14,633 |  | -88.3 | 14,633 |
| *Euphonia chlorotica* | 81,143 |  | -75.2 | 20,157 |  | -0.1 | 20,131 |  | -0.4 | 20,051 |  | -1.0 | 19,860 |  | -37.2 | 12,468 |  | -84.6 | 12,468 |
| *Euterpe edulis* | 2,417 |  | -59.7 | 973 |  | 0.0 | 973 |  | -22.4 | 755 |  | 0 | 755 |  | -34.3 | 495 |  | -79.5 | 495 |
| *Megarhynchus pitangua* | 346,785 |  | -80.0 | 69,217 |  | 0.0 | 69,186 |  | -0.7 | 68,726 |  | -1.3 | 67,811 |  | -51.5 | 32,915 |  | -90.5 | 32,914 |
| *Myiodynastes solitarius* | 156,714 |  | -75.0 | 39,253 |  | -42.9 | 22,427 |  | -1.0 | 22,211 |  | -1.1 | 21,967 |  | -36.8 | 13,879 |  | -91.1 | 13,879 |
| *Penelope superciliaris* | 6,496 |  | -47.4 | 3419 |  | -8.9 | 3,115 |  | -1.7 | 3,062 |  | -27.2 | 2,230 |  | -21.5 | 1,751 |  | -73.0 | 1,751 |
| *Pitangus sulphuratus* | 140,4619 |  | -84.8 | 21,3645 |  | -14.7 | 182,207 |  | -5.7 | 171,898 |  | -0.6 | 170,831 |  | -80.5 | 16,668 |  | -98.8 | 16,668 |
| *Ramphastos toco* | 47,577 |  | -69.9 | 14,330 |  | -0.4 | 14,271 |  | -2.0 | 13,990 |  | -0.7 | 13,894 |  | -40.5 | 8,265 |  | -82.6 | 8,265 |
| *Ramphocelus carbo* | 127,577 |  | -76.7 | 29,762 |  | -0.1 | 29,747 |  | -1.1 | 29,417 |  | -1.3 | 29,021 |  | -50.9 | 14,247 |  | -88.8 | 14,247 |
| *Saltator similis* | 39,984 |  | -74.0 | 10,414 |  | -0.5 | 10,360 |  | -1.0 | 10,256 |  | -1.1 | 10,142 |  | -35.8 | 6,515 |  | -83.7 | 6,515 |
| *Sapajus nigritus* | 3,546 |  | -42.4 | 2,044 |  | -0.3 | 2,038 |  | -35.7 | 1,311 |  | -8.0 | 1,206 |  | -19.2 | 975 |  | -72.5 | 975 |
| *Stilpnia cayana* | 97,208 |  | -76.1 | 23,191 |  | -31.8 | 15,815 |  | 0.0 | 15,815 |  | -0.5 | 15,741 |  | -43.7 | 8,857 |  | -90.9 | 8,857 |
| *Tachyphonus coronatus* | 51,543 |  | -79.7 | 10,446 |  | -0.2 | 10,421 |  | -1.0 | 10,313 |  | -1.3 | 10,183 |  | -45.5 | 5,547 |  | -89.2 | 5,547 |
| *Tangara palmarum* | 430,083 |  | -80.8 | 82,398 |  | 0.0 | 82,389 |  | -0.6 | 81,888 |  | -0.6 | 81,428 |  | -59.5 | 7,231 |  | -98.3 | 7,231 |
| *Tangara sayaca* | 235,253 |  | -85.2 | 34,861 |  | -0.3 | 34,773 |  | -0.9 | 34,457 |  | -0.8 | 34,183 |  | -44.9 | 18,847 |  | -92.0 | 18,847 |
| *Turdus albicollis* | 37,431 |  | -76.3 | 8,876 |  | -2.8 | 8,624 |  | -1.2 | 8,517 |  | -2.8 | 8,282 |  | -36.7 | 5,240 |  | -86.0 | 5,240 |
| *Turdus amaurochalinus* | 124,027 |  | -80.2 | 24,563 |  | -0.8 | 24,372 |  | -0.9 | 24,159 |  | -2.1 | 23,648 |  | -37.6 | 14,769 |  | -88.1 | 14,769 |
| *Turdus flavipes* | 24,044 |  | -77.9 | 5,311 |  | -0.5 | 5,287 |  | -7.1 | 4,913 |  | -0.3 | 4,900 |  | -43.2 | 2,782 |  | -88.4 | 2,782 |
| *Turdus leucomelas* | 173,683 |  | -81.9 | 31,485 |  | -1.3 | 31,090 |  | -0.8 | 30,846 |  | -0.8 | 30,592 |  | -49.2 | 15,546 |  | -91.0 | 15,546 |
| *Turdus rufiventris* | 235,253 |  | -85.6 | 33,812 |  | -1.7 | 33,225 |  | -2.3 | 32,470 |  | -0.6 | 32,288 |  | -46.5 | 17,234 |  | -92.7 | 17,234 |
| *Turdus subalaris* | 3,086 |  | -60.6 | 1,215 |  | -1.2 | 1,200 |  | -0.3 | 1,196 |  | -0.9 | 1,185 |  | -18.6 | 964 |  | -68.8 | 964 |

**Supplementary Table 10 |** **Predictor variables used in species distribution models (SDMs).** Environmental predictors included bioclimatic and soil variables; the latter were applied only in plant SDMs.

| Category | Environmental predictors | Abbreviation | Unit |
| --- | --- | --- | --- |
| Bioclimatic variables (source: CHELSA version 2.1) | Mean annual air temperature | bio1 | °C |
|  | Mean diurnal air temperature range | bio2 | °C |
|  | Isothermality | bio3 | °C |
|  | Temperature seasonality | bio 4 | °C/100 |
|  | Mean daily maximum air temperature of the warmest month | bio5 | °C |
|  | Mean daily minimum air temperature of the coldest month | bio6 | °C |
|  | Annual range of air temperature | bio7 | °C |
|  | Mean daily mean air temperatures of the wettest quarter | bio8 | °C |
|  | Mean daily mean air temperatures of the driest quarter | bio9 | °C |
|  | Mean daily mean air temperatures of the warmest quarter | bio10 | °C |
|  | Mean daily mean air temperatures of the coldest quarter | bio11 | °C |
|  | Annual precipitation amount | bio12 | kg m^-2^ year^-1^ |
|  | Precipitation amounts of the wettest month | bio13 | kg m^-2^ month^-1^ |
|  | Precipitation amounts of the driest month | bio14 | kg m^-2^ month^-1^ |
|  | Precipitation seasonality | bio15 | kg m^-2^ |
|  | Mean monthly precipitation amount of the wettest quarter | bio16 | kg m^-2^ month^-1^ |
|  | Mean monthly precipitation amount of the driest quarter | bio17 | kg m^-2^ month^-1^ |
|  | Mean monthly precipitation amount of the warmest quarter | bio18 | kg m^-2^ month^-1^ |
|  | Mean monthly precipitation amount of the coldest quarter | bio19 | kg m^-2^ month^-1^ |
|  | Growing degree days heat sum above 0°C | gdd0 | °C |
|  | Growing season length TREELIM | gsl | number of days |
|  | Accumulated precipitation amount on growing season days TREELIM | gsp | kg m^-2^ gsl^-1^ |
|  | Mean temperature of the growing season TREELIM | gst | °C |
|  | Net primary productivity | npp | g C m^−2^ yr^-1^ |
| Soil variables (source: [SoilGrids](http://www.soilgrids.org) v2.0) | Bulk density of the fine earth fraction | bdod | cg cm^−3^ |
|  | Cation Exchange Capacity of the soil | cec | mmol(+) kg^-1^ |
|  | Clay (< 0.002 mm) in fine earth | clay | % |
|  | Sand (> 0.05 mm) in fine earth | sand | % |
|  | Organic carbon density | ocd | hg m^-3^ |
|  | Ph (H_2_O) | phh2o | - |
|  | Silt (0.002-0.05 mm) in fine earth | silt | % |
|  | Soil organic carbon in fine earth | soc | g kg^-1^ |

**Supplementary Table 11 | Components and performance of the ensemble species distribution models (SDMs).** Shown are the number of presence records, environmental predictors, and model performance metrics (AUC = Area Under the Receiver Operating Characteristic Curve; TSS = True Skill Statistic; mean ± SD across 45 replications) for the silver cecropia (*Cecropia hololeuca*), jussara palm (*Euterpe edulis*), and their frugivores. Threshold values (Th) used to convert habitat suitability to binary maps are also provided. Predictor abbreviations are detailed in Supplementary Table 10.

| Taxonomic name |  | # records |  | AUC |  | TSS |  | Th | Environmental predictors | | | | | | |  |
| --- | --- | --- | --- | --- | --- | --- | --- | --- | --- | --- | --- | --- | --- | --- | --- | --- |
| *Cecropia hololeuca* | | 109 |  | 0.91 ± 0.08 |  | 0.80 ± 0.13 |  | 0.64 | BIO3 | BIO4 | BIO8 | GSL | NPP | SAND | SILT | SOC |
| *Euterpe edulis* | | 496 |  | 0.93 ± 0.02 |  | 0.78 ± 0.07 |  | 0.66 | BIO2 | BIO4 | BIO8 | BIO13 | BIO19 | GSL | SAND | SOC |
| *Baryphthengus ruficapillus* | | 1550 |  | 0.95 ± 0.01 |  | 0.80 ± 0.05 |  | 0.68 | BIO3 | BIO7 | BIO8 | BIO18 | GSL | NPP |  |  |
| *Callithrix penicillata* | | 505 |  | 0.93 ± 0.03 |  | 0.75 ± 0.07 |  | 0.61 | BIO3 | BIO7 | BIO8 | BIO15 | BIO19 | GSL |  |  |
| *Cyclarhis gujanensis* | | 28662 |  | 0.81 ± 0.07 |  | 0.5 ± 0.13 |  | 0.58 | BIO2 | BIO3 | BIO8 | BIO9 | BIO13 | BIO14 | BIO18 | BIO19 |
| *Dacnis cayana* | | 14633 |  | 0.87 ± 0.04 |  | 0.60 ± 0.08 |  | 0.60 | BIO2 | BIO3 | BIO8 | BIO13 | BIO14 | BIO18 | BIO19 | GSL |
| *Euphonia chlorotica* | | 12469 |  | 0.81 ± 0.08 |  | 0.5 ± 0.15 |  | 0.61 | BIO2 | BIO3 | BIO8 | BIO13 | BIO14 | BIO18 | BIO19 | GSL |
| *Megarynchus pitangua* | | 32915 |  | 0.86 ± 0.50 |  | 0.59 ± 0.09 |  | 0.47 | BIO2 | BIO3 | BIO8 | BIO13 | BIO14 | BIO18 | BIO19 | GSL |
| *Myiodynastes solitarius* | | 13879 |  | 0.82 ± 0.06 |  | 0.52 ± 0.11 |  | 0.62 | BIO2 | BIO3 | BIO8 | BIO13 | BIO14 | BIO18 | BIO19 | GSL |
| *Penelope superciliaris* | | 1751 |  | 0.85 ± 0.05 |  | 0.57 ± 0.09 |  | 0.63 | BIO2 | BIO8 | BIO13 | BIO14 | BIO18 | BIO19 | GSL |  |
| *Pitangus sulphuratus* | | 33244 |  | 0.76 ± 0.07 |  | 0.57 ± 0.14 |  | 0.65 | BIO2 | BIO3 | BIO8 | BIO14 | BIO18 | BIO19 | GSL |  |
| *Ramphastos toco* | | 8265 |  | 0.90 ± 0.03 |  | 0.69 ± 0.07 |  | 0.66 | BIO2 | BIO3 | BIO8 | BIO13 | BIO14 | BIO18 | BIO19 | GSL |
| *Ramphocelus carbo* | | 14246 |  | 0.84 ± 0.07 |  | 0.54 ± 0.15 |  | 0.54 | BIO2 | BIO3 | BIO8 | BIO13 | BIO14 | BIO18 | BIO19 | GSL |
| *Saltator similis* | | 6515 |  | 0.92 ± 0.02 |  | 0.72 ± 0.05 |  | 0.69 | BIO2 | BIO3 | BIO8 | BIO9 | BIO18 | GSL | NPP |  |
| *Sapajus nigritus* | | 975 |  | 0.94 ± 0.02 |  | 0.79 ± 0.04 |  | 0.75 | BIO2 | BIO7 | BIO8 | BIO18 | GSL | NPP |  |  |
| *Stilpnia cayana* | | 8857 |  | 0.87 ± 0.07 |  | 0.60 ± 0.14 |  | 0.65 | BIO2 | BIO3 | BIO8 | BIO13 | BIO14 | BIO18 | BIO19 | GSL |
| *Tachyphonus coronatus* | | 5547 |  | 0.95 ± 0.01 |  | 0.84 ± 0.02 |  | 0.71 | BIO3 | BIO7 | BIO8 | BIO9 | BIO18 | GSL | NPP |  |
| *Tangara palmarum* | | 32993 |  | 0.84 ± 0.07 |  | 0.56 ± 0.14 |  | 0.69 | BIO2 | BIO3 | BIO8 | BIO13 | BIO14 | BIO19 | GSL |  |
| *Tangara sayaca* | | 18846 |  | 0.87 ± 0.06 |  | 0.60 ± 0.1 |  | 0.75 | BIO2 | BIO3 | BIO8 | BIO9 | BIO13 | BIO19 | GSL |  |
| *Turdus albicolis* | | 5240 |  | 0.89 ± 0.03 |  | 0.66 ± 0.07 |  | 0.68 | BIO2 | BIO3 | BIO8 | BIO9 | BIO14 | BIO18 | BIO19 | NPP |
| *Turdus amaurochalinus* | | 14765 |  | 0.88 ± 0.04 |  | 0.66 ± 0.06 |  | 0.73 | BIO2 | BIO3 | BIO8 | BIO9 | BIO18 | BIO19 | GSL |  |
| *Turdus flavipes* | | 2783 |  | 0.96 ± 0.04 |  | 0.84 ± 0.04 |  | 0.66 | BIO2 | BIO3 | BIO8 | BIO15 | BIO18 | GSL | NPP |  |
| *Turdus leucomelas* | | 15546 |  | 0.85 ± 0.07 |  | 0.57 ± 0.14 |  | 0.53 | BIO 2 | BIO3 | BIO8 | BIO13 | BIO14 | BIO19 | BIO19 | GSL |
| *Turdus rufiventris* | | 17287 |  | 0.90 ± 0.03 |  | 0.70 ± 0.06 |  | 0.72 | BIO2 | BIO3 | BIO8 | BIO9 | BIO18 | BIO19 | GSL |  |
| *Turdus subalaris* | | 964 |  | 0.94 ± 0.01 |  | 0.81 ± 0.03 |  | 0.76 | BIO2 | BIO7 | BIO8 | BIO9 | BIO12 | BIO14 | NPP | GSL |
